# Characterizing altruistic motivation in potential volunteers for SARS-CoV-2 challenge trials

**DOI:** 10.1371/journal.pone.0275823

**Published:** 2022-11-02

**Authors:** Abigail A. Marsh, Monica Magalhaes, Matthew Peeler, Sophie M. Rose, Thomas C. Darton, Nir Eyal, Josh Morrison, Seema K. Shah, Virginia Schmit

**Affiliations:** 1 Department of Psychology, Georgetown University, Washington, DC, United States of America; 2 Center for Population-Level Bioethics, Rutgers University, New Brunswick, New Jersey, United States of America; 3 Department of Mathematics, Rowan-Cabarrus Community College, Salisbury, North Carolina, United States of America; 4 Department of Epidemiology, Johns Hopkins Bloomberg School of Public Health, Baltimore, Maryland, United States of America; 5 Department of Infection, Immunity and Cardiovascular Disease, University of Sheffield, Sheffield, United Kingdom; 6 Department of Health Behavior, Society and Policy, Rutgers School of Public Health, Piscataway, New Jersey, United States of America; 7 Department of Philosophy, Rutgers University, New Brunswick, New Jersey, United States of America; 8 1Day Sooner, Claymont, Delaware, United States of America; 9 Smith Child Health Outcomes, Research and Evaluation Center, Lurie Children’s Hospital, Chicago, Illinois, United States of America; 10 Department of Pediatrics, Northwestern University Feinberg School of Medicine, Chicago, Illinois, United States of America; Sapienza, University of Rome, ITALY

## Abstract

In human challenge trials (HCTs), volunteers are deliberately infected with an infectious agent. Such trials can be used to accelerate vaccine development and answer important scientific questions. Starting early in the COVID-19 pandemic, ethical concerns were raised about using HCTs to accelerate development and approval of a vaccine. Some of those concerns pertained to potential exploitation of and/or lack of truly informed consent from volunteers. Specific areas of concern arose around individuals who may be unusually risk-seeking or too economically vulnerable to refuse the payments these trials provide, as opposed to being motivated primarily by altruistic goals. This pre-registered study is the first large-scale survey to characterize people who, early in the pandemic, expressed interest and intention to volunteer to participate in COVID-19 HCTs. We found that individuals expressing interest in SARS-CoV-2 HCTs exhibit consistently altruistic motivations without any special indication of poor risk perception or economic vulnerability. In finding that, early in the pandemic, COVID-19 HCTs were able to attract volunteers whose values align with the nature of these trials, and who are not unusually vulnerable to exploitation, this study may allay some ethical concerns about the volunteers interested in participating in such trials.

## Introduction

The ongoing COVID-19 pandemic presents extraordinary threats to public health and human welfare. Economic and social recovery will require further development and testing of prevention strategies, including vaccines that are easier to provide, store, and deliver. More research on various vaccine dosing regimens is required, and vaccines may need to be updated to keep pace with emerging variants [1]. Human challenge trials (HCTs), in which volunteers are deliberately infected (or “challenged”) with an infectious agent to test the efficacy of vaccine candidates, are among the most efficient and scientifically powerful approaches to testing vaccines and learning about early disease processes [2]. Well-designed HCTs can speed the development of improved vaccines and dosing regimens by selecting the most promising candidates to prioritize for further testing [3–8].

A salient feature of HCTs that sometimes makes them controversial is that HCTs require participants to consent to undergo an intervention—deliberate infection with a virus—that is expected to be harmful. By contrast, in clinical studies that aim to test an intervention’s effectiveness participants consent to an intervention that has a chance of being beneficial. In this respect, HCTs are similar to phase 1 trials, in which safety, side effects, and other features of a new interventions are tested in healthy volunteers. In effectiveness trials, harm to participants, while possible, is generally an unintended side-effect.

The potential benefits from HCTs are largely societal, as participants themselves have little prospect of direct benefit. By contrast, the risks and burdens of HCTs—including infection-related risks, prolonged periods of biocontainment, and potential side effects from trial vaccines or treatments—fall largely on volunteers [9]. These risks and burdens (which are heightened by uncertainty about COVID-19 disease outcomes) coupled with the absence of obvious direct benefits for volunteers have led some bioethicists to suggest that HCTs using the novel coronavirus may be unethical [10–12]. Some commentators have worried (beginning long before the COVID-19 pandemic) that HCTs might attract healthy volunteers who are vulnerable to undue inducement or exploitation. This concern may be grounded in the literature about healthy volunteers who participate in phase 1 trials, where the frequent recruitment of financially desperate, predominantly poor and ethnic minority, often uninsured participants who may enroll in risky trials sequentially as a means to earn a living is often considered exploitative [13–15]. Elliott and Abadie [14] specifically cast doubt on claims that participants in phase 1 trials can plausibly be called “altruistic” or “volunteers”. A related but distinct worry is that HCT volunteers may have problems understanding relevant risks, which might invalidate their consent or result in their exploitation [16, 17]. These concerns, alongside scientific uncertainty around COVID-19, have complicated decision-making about HCTs that could have speeded up COVID-19 vaccine research early in the pandemic [18]. As a result of such concerns, a COVID-19 HCT has been completed in the UK [19] only far later than it might have otherwise been done, when the uncertainty around the risks and benefits of these trials was very different than it had been at the time of this survey. This survey of early-pandemic intended HCT volunteers, assessing altruistic traits and indicators of vulnerability, may help preempt similar concerns, and resulting delays to highly socially beneficial research, in future pandemics.

Direct benefits to participants are not required for human subjects research to be considered ethical [20]. Instead, the totality of the benefits—including benefits to others—should be sufficient to justify the risks. HCTs should also be designed to expose participants to as few risks as possible, and participants must be able to provide valid informed consent [21]. This requires providing volunteers with the opportunity to evaluate the risks, benefits, and alternatives to any intervention to ensure that it reflects their goals, preferences, and values [22].

Given the altruistic nature of HCT participation—with volunteers required to take on personal risks and costs to achieve societal benefits—it would be ideal, from an ethical perspective, if volunteers demonstrated highly altruistic goals, values, and preferences. We do not take a position on whether altruistic motivation is required for HCTs to be permissible, or on whether participants’ financial motivation (either alone or in addition to altruistic motivation) renders a study impermissible. However, participants in various other types of research are known to have mixed motivations (including both altruistic and non-altruistic motivations) [23]. Our stance is that it would be ideal if HCT volunteers had altruistic motivations in line with the goals of this type of research, which is primarily conducted to benefit others, as altruistic motivation would most clearly support understanding of the HCT goals and implications.

To date, few studies have examined why healthy volunteers consent to research with net risks and burdens to themselves, or whether their goals and values are compatible with ethical participation [24–30]. Stunkel and Grady’s [23] meta-analysis of the literature on the motivations of healthy volunteers mainly investigated studies conducted in the United States and Europe. They found that, while financial motivation is the primary motivation for participation in most of the studies surveyed, most participants had mixed motivations that included helping others and contributing to science. They further found that the medical risk of a trial is the main disincentive to participate.

There have been few prior studies [25, 26, 31–33] that investigated volunteer motivations to participate in HCTs specifically. Two of these [31, 33] appear to survey participants of the same challenge trial. We included both for completeness even though both reports appear to refer to the same study. Generally, these studies also found that financial motivation is the primary motivation for participation. Two of these studies [25, 31] also mentioned access to health checks and screening tests as motivating factors. However, four of these studies [25, 31–33] surveyed HCT participants in developing countries, so their samples are also significantly unlike the sample surveyed for this report, which is mostly made up of intended volunteers from high-income countries. Kraft et al. [26] interviewed participants in controlled human malaria studies, which involves use of an established, safe challenge model that is very different from a challenge study with an emerging infectious disease. These studies [25, 26, 31–33] of HCT participants all also found altruistic motivation among the main motivating factors, whether from a desire to contribute to science, to contribute to the health of developing countries, or to contribute to the health of participants’ own communities. No study to date has described the motivations of volunteers who have declared their willingness to participate in HCTs with the novel coronavirus.

To assess whether a group of individuals who proactively declared their interest in volunteering to participate in a COVID-19 HCT is in fact altruistically motivated (as, in our view, would be ideal), we conducted the first large-scale evaluation of characteristics of volunteers expressing interest in and intention to participate in COVID-19 HCTs, should they become available (henceforth referred to as “volunteers” for brevity, even though they would not have volunteered for a COVID-19 HCT at the time of the survey, as none had recruited; and are unlikely to ever have done so, given how few COVID -19 HCTs ever took place). Volunteers were recruited through the non-profit advocacy organization 1Day Sooner (https://www.1daysooner.org/). 1Day Sooner was created in April 2020 to accelerate the deployment of effective vaccines by supporting preparation efforts for COVID-19 HCTs and to advocate on behalf of potential COVID-19 HCT volunteers. It curates the only centralized international database of volunteers who have indicated their willingness to partake in COVID-19 HCTs.

We hypothesized that COVID-19 HCT volunteerism reflects heightened altruistic values and preferences. In light of concerns that HCTs may attract participants who are unusually insensitive to risk or who are in dire economic need [10, 11, 26], we also tested the alternate hypotheses that COVID-19 HCTs attract participants who engage in elevated risk behaviors (including specifically health and safety related risk behaviors) or those who are economically or otherwise vulnerable to exploitation. Either of these issues could raise concerns about the ethical permissibility of COVID-19 HCTs. To test these hypotheses, we conducted a pre-registered (https://osf.io/fqyrb) study in which we measured altruistic motivation, values, and behavior; risk preferences and behaviors, and sociodemographic variables in potential COVID-19 HCT volunteers.

## Methods

### Participants

**Fig 1:**
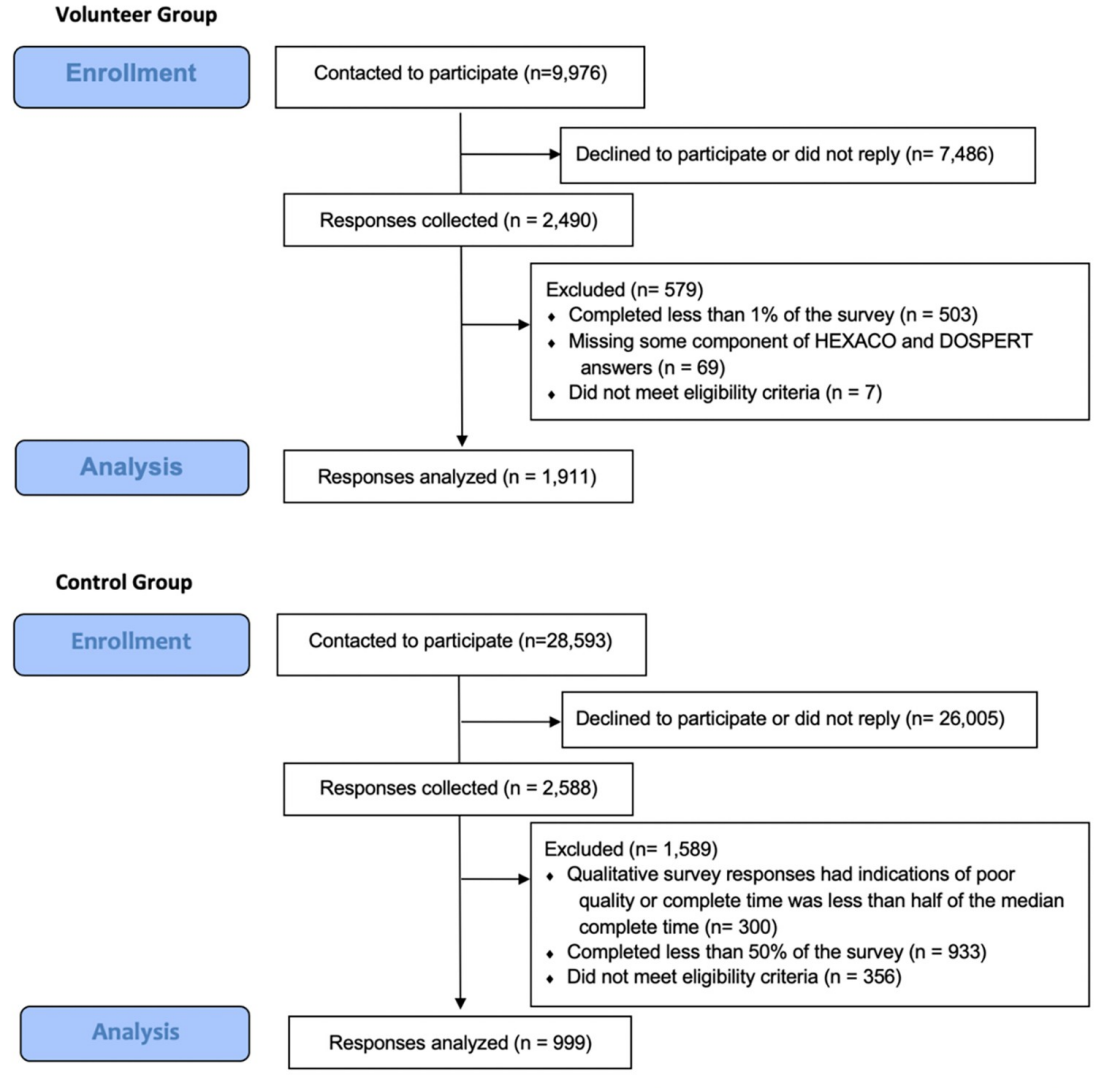
CONSORT diagram of volunteer and control group enrollment and analysis. 9,976 volunteers from the 1Day Sooner database who had indicated they were interested in contributing to further research were contacted to participate in this study. Of these, 7,486 volunteers did not reply or declined to participate. The remaining 2,490 volunteers completed the survey via the Qualtrics platform (25% response rate). 579 of these responses were ultimately excluded from the final analysis due to failure to complete sufficient portions of the survey, missing data, or submitting a birth date that indicated they were under 18 years of age. The remaining 1,911 responses were analyzed for inclusion in this report.

2,910 individuals completed a 45-minute online survey that included indices of altruistic motivation, values, and behavior; an assessment of risk preferences and behaviors; and a survey of sociodemographic variables. Questions presented to the volunteer and control group are available in S4 File. The sample included 1,911 individuals, all of whom had previously indicated their interest in volunteering for COVID-19 HCTs prior to May 29, 2020, as well as 999 controls. Participant demographic characteristics are described in Table 1. Sample size was limited by the budget constraints of the non-profit advocacy organization 1Day Sooner. Volunteers were recruited through 1Day Sooner, and at the time of survey, none of these individuals had yet participated in a COVID-19 HCT (or had any opportunity to do so, as none had ever recruited participants at that point). Volunteers who had declared their interest in volunteering and provided their contact information as well as their interest in participating in research were recruited via email (Fig 1). Volunteers in the database were excluded from sampling if they were under age 18, responded ‘no’ to a query about wanting to participate in an HCT, declined to share their information with researchers, or declined to provide a response to a query about reasons for participating (open response format). Participants who responded to this question were not included in the analysis for this report if they responded in a language other than English, or if responses were too brief (<5 words) to ascertain fluency in English.

**Table 1:** Participant demographic characteristics.

	Volunteer Group	Control
	(n = 1911)	(n = 999)
	N.o. people (%)	N.o. people (%)
Age		
18–25	252 (13.3)	91 (9.4)
26–35	580 (30.7)	139 (14.4)
36–45	419 (22.2)	195 (20.1)
46–55	297 (15.7)	189 (19.5)
56–65	229 (12.1)	139 (14.4)
66–75	99 (5.2)	180 (18.6)
76+	12 (0.6)	35 (3.5)
Non-responses	23	31
Gender		
Male	1151 (60.4)	436 (43.9)
Female	673 (35.3)	522 (52.5)
Self-identify/Prefer not to say	82 (4.3)	36 (3.6)
Non-response	5	5
Marital Status		
Single (never married)	976 (51.1)	289 (29.1)
Married/Domestic partnership	664 (34.7)	523 (52.6)
Divorced	200 (10.5)	121 (12.2)
Widowed	27 (1.4)	48 (4.8)
Separated	44 (2.3)	13 (1.3)
Non-responses	0	5
Race/Ethnicity		
Selected African (Yes/No (%Yes))	28/1883 (1.5)	95/904 (9.5)
Selected Hispanic (Yes/No (%Yes))	133/1778 (7.0)	59/940 (5.9)
Selected Caucasian (Yes/No (%Yes))	1595/316 (83.5)	706/293 (70.7)
Selected Asian (Yes/No (% Yes))	162/1749 (8.5)	110/889 (12.4)
Selected Native American (Yes/No (% Yes))	32/1879 (1.7)	19/980 (1.9)
Employment (Top 5 categories listed)		
Employed	1009 (52.8)	394 (39.4)
Self-employed/Freelance	197 (10.3)	52 (5.2)
Retired	137 (7.2)	240 (24.0)
Studying	121 (6.3)	36 (3.6)
Unemployed/Looking for work	115 (6.0)	80 (8.0)
Employment Status		
Employed full-time	941 (49.3)	323 (32.6)
Unemployed	728 (38.2)	593 (59.9)
Employed part-time	239 (12.5)	74 (7.5)
Non-responses	3	9
Income (in USD)		
Less than $25K	211 (11.0)	217 (21.8)
$25K-$50K	326 (17.1)	248 (24.9)
$50K-$100K	512 (26.8)	203 (20.4)
$100K-$200K	458 (24.0)	240 (24.1)
Greater than $200K	260 (13.6)	67 (6.7)
Prefer not to say	143 (7.5)	21 (2.1)
Non-responses	1	3
Have health insurance		
Yes	1683 (88.1)	877 (88.1)
No	180 (9.4)	95 (9.5)
Not sure	29 (1.5)	10 (1.0)
Prefer not to say	18 (0.9)	14 (1.4)
Non-responses	1	3
Have Children (Yes/No (% Yes))	549/1360 (28.8)	494/499 (49.7)
Non-responses	2	6
Number in Household (other than self)		
0	611 (32.2)	267 (27.0)
1	656 (34.5)	353 (35.7)
2	260 (13.7)	152 (15.4)
3	228 (12.0)	116 (11.7)
4	89 (4.7)	73 (7.4)
5+	56 (3.0)	29 (2.9)
Non-responses	11	9
Education Level		
Less than High School	4 (0.2)	22 (2.2)
High School Graduate/GED	84 (4.4)	163 (16.3)
Some college, no degree	218 (11.4)	149 (14.9)
Trade/Technical training	65 (3.4)	42 (4.2)
Associate degree	59 (3.1)	102 (10.2)
Bachelor’s degree	684 (35.8)	280 (28.1)
Master’s degree	498 (26.1)	172 (17.3)
Professional degree	119 (6.2)	44 (4.4)
Doctoral degree	180 (9.4)	23 (2.3)
Non-responses	0	2

Control participants were recruited using a private research software company (Qualtrics Panel). Qualtrics identifies individuals through other survey-hosting platforms and the panel is recruited to be reflective of the population distribution captured by the 2019 United States Census. Of 28,593 individuals who were contacted by Qualtrics to participate as control subjects, 26,005 did not reply or declined to participate (9.1% response rate). The 2,588 who responded completed the survey via the Qualtrics platform, and 1,589 were excluded. 999 responses from control participants were analyzed for inclusion in this report.

Inclusion criteria for all participants included age greater than 18 years and demonstrated proficiency in English. All participants who completed the survey were compensated with $5 USD in the form of an electronic gift card. Participants who expressed interest in completing the survey were allotted seven days to complete it at a time of their choosing, and could complete it in more than one sitting if they preferred. Those who did not complete the survey were sent follow-up emails on day 4 and day 6 to give them the opportunity to complete their response. The protocol was approved by the Institutional Review Board at Rutgers University (Study ID: Pro2020001023) and all participants provided electronic informed consent before beginning the survey. All statistical tests for this study were taken from the same sample and are two-tailed tests.

### Survey instruments

Indices of altruistic values and preferences were assessed. First, the volunteer group selected their top three motivations for volunteering from a list of 10 possible motivations drawn from consultations with a panel of HCT researchers and bioethicists (Table 2). Two motivations were primarily altruistic in that they refer to outcomes for entities outside the self (“I wanted to help others and potentially save lives” and “I wanted to contribute to the progress of medicine”); the other 8 reflected various other motivations (e.g., “I wanted to receive the financial reimbursement for participating” or “I was curious about COVID-19”). Controls did not complete this section.

**Table 2:** Volunteer group motivations for participating in human challenge studies.

Motivation	Number (%) rating motivation in the top three reasons for volunteering^1^
I wanted to help others and potentially save lives	1832 (95.9)
I wanted to contribute to the progress of medicine	1513 (79.2)
I feel helpless and this is a way to do something positive	890 (46.6)
Another factor not mentioned	380 (19.9)
I wanted to be part of a clinical trial	348 (18.2)
I am likely to be infected by COVID-19 anyway	282 (14.8)
I was curious about COVID-19	170 (8.9)
I wanted to be guaranteed access to critical care should I be infected with COVID-19	156 (8.2)
I wanted to find out more about my own health	83 (4.3)
I wanted to receive the financial reimbursement for participating	79 (4.1)

^1^ Since volunteers were asked to rate whether the choices above were in their top three reasons, percentages total 300% instead of 100% (with exceptions due to rounding).

Second, participants indicated their prior engagement in various altruistic behaviors that carry varying levels of risk and cost, including blood donation, registering to donate bone marrow, registering to be a deceased organ donor, donating money to charity, and living organ donation.

Third, participants completed two additional instruments assessing personality traits and risk perception: the Brief HEXACO inventory and the DOSPERT scale. The Brief HEXACO inventory is a 24-item measure assessing six dimensions of personality: Honesty-Humility, Emotionality, eXtraversion, Agreeableness, Conscientiousness, and Openness to Experience [34, 35]. Each item is rated on a five-point scale. Unlike five-factor inventories, HEXACO inventories include a subscale (Honesty-Humility) that specifically indexes attitudes and behaviors related to valuation of outcomes for others versus the self (such as exploitation, manipulation, or deceit) and has been consistently linked to prosocial motivation and behavior [36–38]. The DOSPERT scale is a 30-item index that assesses three primary components of risk attitudes (risk-taking, risk-perception and perceived expected benefits) across six broad decision categories: ethical, financial (divided into investment and gambling), health and safety, social, and recreational risks [39]. The *risk-taking scale* assesses respondents’ likelihood of engaging in the risky activity or behavior, the *risk-perception scale* assesses how risky participants perceive each of these activities to be, and the *expected-benefits scale* assesses the degree to which participants perceive each activity to be beneficial. Responses are made using a 7-point scale (1 = Extremely unlikely/Not at all risky/No benefits at all, 7 = Extremely likely/ Extremely risky/Great benefits).

Finally, all participants completed an assessment of socioeconomic and other demographic variables (see Table 1 for a description of these demographic characteristics). Regression models for all analyses included the covariates of age, gender, education level, income and country of residence to control for the potential influence of differences in these characteristics. Age was included as a continuous (scale) variable, centered at the mean age of 43.67 years. Gender was analyzed as a categorical variable, broken down into male (reference), female, self-describe or prefer not to say. Education was analyzed as a categorical variable, broken down as less than high school (reference), high school or equivalent, trade or technical school, associate degree, some college, bachelor’s degree, master’s degree, professional or doctoral degree. Income was analyzed as a categorical variable with six categories—all listed in USD: less than $25k annual household income (reference), $25k-$50k, $50k-$100k, $100k-$200k, greater than $200k, and prefer not to say. Country of residence was dichotomized as non-US (reference) and US.

### HEXACO analysis

Analyses were performed using the standard six established HEXACO dimensions [40], which are reliably linked to variation in social preferences behaviors, with Honesty-Humility in particular linked to variation in prosocial outcomes [36]. We analyzed HEXACO results using a confirmatory factor analysis (CFA) to assess the fit of the HEXACO model to the dataset after listwise removal of missing data (leaving n = 1700 for volunteers, n = 802 controls). Hu and Bentler [41] supported RMSEA measures cutoff scores of < 0.06 as a good fit, 0.07–0.08 as an acceptable fit, 0.08–0.10 as a limited fit, and > 0.10 as unfit, and supported CFI and TLI cutoffs of > 0.95 as a sufficient fit, 0.90–0.95 as an acceptable fit, and < 0.90 as a poor fit. The sample’s HEXACO responses generally showed limited to poor fit ($\frac{\chi^{2}}{df}=24.014,CFI=0.544,TFI=0.469,RMSEA=0.096$), (see S1 Table for a comparison of the CFA factor loadings and S2 Table for the original HEXACO dimensions by question). The results observed herein are consistent with observations by other researchers using this instrument [42, 43]. Some researchers attribute potential poor fit of the model to various inconsequential issues [44], while others are more concerned about poorly fitting models and argue that social desirability and acquiescence bias should be included in the model [45]. Still others suggest a poor fit of the model simply reflects the limited validity of these personality factors [46–48].

Factor loading scores on each HEXACO dimension were then calculated and compared across volunteer and control groups using an ANCOVA model controlling for age, income, education level, country of residence and gender. Subsequent analyses were conducted to determine the likelihood of participants being in the volunteer or control group based on their HEXACO scores. These analyses were initially conducted using a multivariate logistic regression containing only the six HEXACO dimensions as independent variables, and membership in the volunteer group as the dependent variable. An additional multivariate analysis including the demographic covariates of age, gender, education level, income, and US residency was then conducted.

### DOSPERT analysis

Analyses using the DOSPERT scale were performed using the six original DOSPERT domains: Ethical, Financial—Investment, Financial—Gambling, Health/Safety, Recreational and Social [39, 49]. We analyzed DOSPERT data using CFA to assess the fit of the data to the three DOSPERT domains. A separate CFA was performed on each component of the DOSPERT scale (risk-taking, risk perception, and perceived expected benefits) with all three DOSPERT scales showing a marginally poor to acceptable level of fit (DOSPERT Preference: $\frac{\chi^{2}}{df}=9.894,CFI=0.880,TFI=0.866,RMSEA=0.060$, DOSPERT Risk: $\frac{\chi^{2}}{df}=13.091,CFI=0.859,TFI=0.843,RMSEA=0.070$, DOSPERT Benefit: $\frac{\chi^{2}}{df}=13.393,CFI=0.880,TFI=0.866,RMSEA=0.070$) (See S3–S5 Tables for a comparison of the CFA factor loadings for risk-taking behavior, risk perception, and expected benefits respectively, and S6 Table for the original DOSPERT dimensions by question).

Factor loading scores for risk behaviors and evaluations were then calculated and compared across the volunteer and control groups using an ANCOVA model that included an additional covariate for age, and included the categorical variables of income, education level, gender, and country of residence as fixed effects to control for the potential role of demographic differences between volunteers and controls.

### Statistical analysis

All statistical analyses were performed using the Statistical Product and Service Solutions statistical software package, version 27.0, and the Amos statistical software package, version 27.0 (SPSS, IBM Corporation, Armonk, NY).

## Results

### Socio-demographic variables

Most volunteers (66.2%) were between 18 and 45 years of age, identified as non-Hispanic white (78.5%), and had a bachelor’s degree or higher (77.4%). A majority reported residing in the United States (81.5%), followed by Canada (7.6%), the United Kingdom (2.3%) and Germany (1.0%). Most volunteers had either private health insurance or access to healthcare through publicly-funded health systems (88.1%). Approximately one in three volunteers (32.0%) lived alone, and a similar proportion (34.4%) lived with only one other person. 28.7% of volunteers had at least one child. Half of volunteers were employed full-time (50.8%) and most (71.9%) reported an annual household income greater than $50,000 USD. Of the total, 213 (11.5%) reported an annual household income less than $25,000 USD; 23% of these (49/213) were students.

As shown in Table 1, there are noteworthy differences in demographic and socio-economic factors between the sample of volunteers and controls. This is to be expected: a group who became informed early in the pandemic about HCTs and 1DaySooner’s initiative, and chose to proactively sign up, are not a random or representative slice of the population. Comparing the two groups, more volunteers were male relative to the general population and controls. Only 35.3% of volunteers self-identified as female (3.2% self-identified as non-binary or transgender, and 1.1% did not specify their gender), whereas the control group, which was recruited to reflect general population demographics, had a more equal gender distribution. Volunteers were generally younger and more educated. Volunteers were also wealthier; assuming equal distribution within income categories, 61.9% of volunteers were above the U.S. median income ($68,703 annually), compared to 45.7% of the control group. Of volunteers, 11.8% fell below the U.S. poverty line ($26,172 annually for a family of four), compared to 23.0% of controls [50]. Volunteers and controls reported equal levels of health insurance.

### Altruistic values and preferences

Following our pre-registered analysis plan, we conducted an exploratory factor analysis on responses to the 10 motivations for volunteering (see S1 File), which returned a three factor solution, with one factor comprising the two altruistic motivations. The percentages of participants who selected each of the motivations were calculated (Table 2). The two altruistic motivations were the only options selected by the majority of volunteers. Both altruistic motivations were selected by over three-quarters of volunteers (“I wanted to help others and potentially save lives” (95.9%) and “I wanted to contribute to the progress of medicine” (79.2%)). The third most highly ranked choice (“I feel helpless and this is a way to do something positive” (46.6%)) was selected by a minority of volunteers, as were the remaining options.

**Fig 2:**
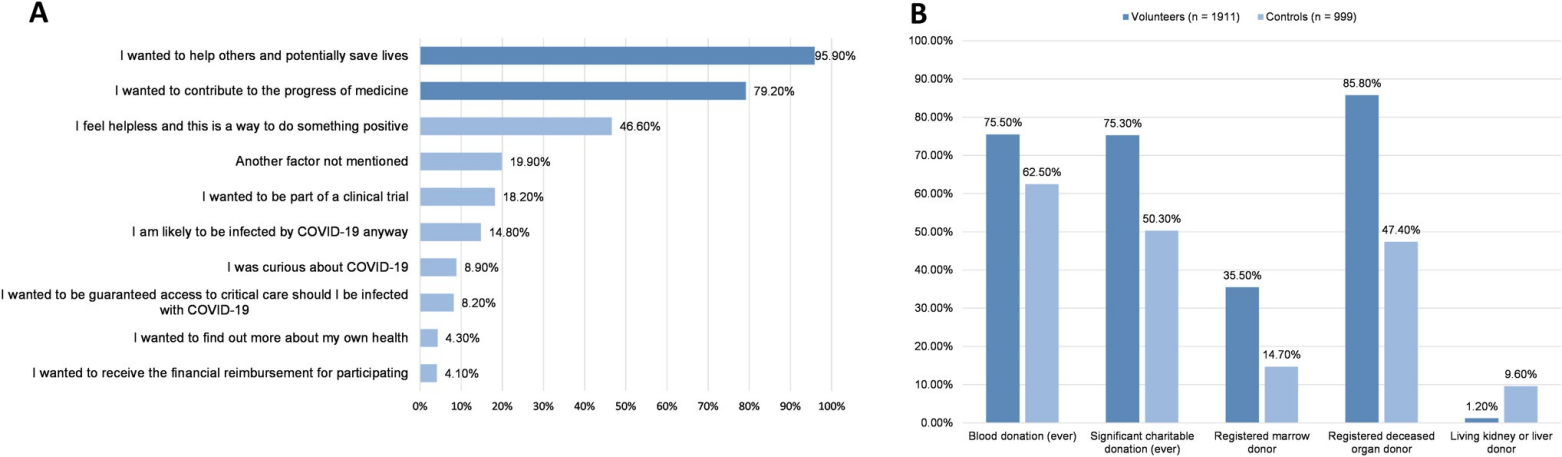
Volunteer group motivations for participating in human challenge studies and comparison of engagement in altruistic behaviors by volunteer vs. control group. (A) Participants in the volunteer group were asked to indicate their top three motivations for participating in a COVID-19 challenge trial from a list of ten options. Selections were not ranked, and total percentages add to 300% because each participant selected 3 options. The two most commonly selected options were “I wanted to help others and potentially save lives” (95.9%) and “I wanted to contribute to the progress of medicine” (79.2%). (B) Participants in volunteer and control groups were surveyed on their engagement with a range of altruistic behaviors, including blood donation, significant charitable donations and organ/marrow donor status. Volunteers were significantly more likely than controls to have participated in all but one of the altruistic behaviors.

We next conducted chi-square tests to compare volunteers’ and controls’ prior engagement in altruistic behavior and found that volunteers were more likely than controls to have participated in all but one of these behaviors (Fig 2). More volunteers reported having previously donated blood (V: 75.5%, C: 62.5%, **χ**^2^(1) = 54.020, p<0.001), having donated significant amounts of money to charity (V: 75.3%, C: 50.3%, **χ**^2^(1) = 175.374, p<0.001), registering as a bone marrow donor (V: 35.5%, C: 14.7%, **χ**^2^(1) = 124.284, p<0.001) or being a registered deceased organ donor (V: 85.8%, C: 47.4%, **χ**^2^(1) = 460.221, p<0.001). More controls reported being living kidney or liver donors (V: 1.2%, C: 9.6%, **χ**^2^(1) = 116.813, p<0.001), but positive response rates for controls were implausibly high given the overall prevalence of living organ donation (per capita prevalence < 1 in 100,000), suggesting results for this question may not be reliable.

We next compared volunteer and control groups along each HEXACO fitted score using an ANCOVA model, controlling for age, income, education level, gender, and country of residence. Effect sizes were calculated using eta-squared (η^2^) values [51], with effect sizes <0.01 considered trivial, effect sizes 0.01–0.06 small, effect sizes 0.06–0.14 medium, and effect sizes >0.14 large. Average scores for volunteers were significantly higher than controls on all but one of the HEXACO dimensions, with a large effect size obtained for both Honesty-Humility (V: 1.00, C: 0.86, p<0.001, η^2^ = 0.156) and eXtraversion (V: 3.69, C: 3.17, p <0.001, η^2^ = 0.141). In contrast, HCT volunteers scored lower on Emotionality, but this effect size was medium (V: -0.90, C: -0.60, p<0.001, η^2^ = 0.090) (Table 3).

**Table 3:** Comparisons of HEXACO fitted scores by volunteer vs. control group membership.

	Volunteer Group		Control		p-value	η2
	(n = 1700)		(n = 802)			
	*Mean (SE)*	*95% CI*	*Mean (SE)*	*95% CI*		
***Honesty-Humility (H)***	0.997 (0.003)	0.991, 1.004	0.859 (0.005)	0.849, 0.869	<0.001	0.156
***Emotionality (E)***	-0.904 (0.010)	-0.924, -0.885	-0.603 (0.015)	-0.633, -0.572	<0.001	0.090
***eXtraversion (X)***	3.689 (0.013)	3.663, 3.715	3.171 (0.021)	3.131, 3.211	<0.001	0.141
***Agreeableness (A)***	2.342 (0.011)	2.320, 2.364	1.944 (0.017)	1.911, 1.978	<0.001	0.123
***Conscientiousness (C)***	1.167 (0.005)	1.156, 1.177	1.004 (0.008)	0.988, 1.020	<0.001	0.093
***Openness to Experience (O)***	2.097 (0.009)	2.078, 2.115	1.815 (0.014)	1.787, 1.843	<0.001	0.089

Marginal means by group are the means for the two groups controlled for all covariates (assuming the mean age of the sample of 43.58). P-values were calculated using F-tests. Effect sizes were calculated using eta-squared (η^2^), with cutpoints for small, medium, and large effects defined as 0.01, 0.06, and 0.14 respectively.

#### Volunteer-control logistic regression results

We used logistic regression analyses to predict the likelihood of a participant being in the volunteer group based solely on HEXACO outcomes. OR refers to the odds ratio, and Cohen’s d is calculated from the odds ratio using the formula d = ln(OR) * sqrt(3)/pi [52, 53]. Standard levels for Cohen’s d [51] are as follows: 0.2 is a trivial effect size—0.2–0.5 is small—0.5–0.8 is medium, and greater than 0.8 is large.

Results indicated that HCT volunteer status was most strongly predicted by Honesty-Humility (OR: 4550.745, 95% CI: 914.088, 22655.685, d = 4.644) when controlling for the five other HEXACO dimensions. Openness to Experience was the next most strongly associated with volunteer group membership (OR: 8.612, 95% CI: 6.075, 12.209, d = 1.187). Emotionality (OR: 0.244, 95% CI: 0.105, 0.570, d = 0.778), eXtraversion (OR: 0.623, 95% CI: 0.449, 0.866, d = 0.261) and Conscientiousness (OR: 0.021, 95% CI: 0.005, 0.087, d = 2.130) were negatively predictive of volunteer status (i.e. more likely to predict control status). Agreeableness was not a statistically significant predictor. This model had a Cox & Snell R^2^ = 0.239 and a -2 log-likelihood value of 2455.368. See S2 File for a brief discussion on the large observed odds ratio values.

We then added demographic covariates to the above model, including age, gender, education level, income, and country of residence to control for the potential influence of these differences between volunteers and controls. When controlling for the five other HEXACO dimensions, the results again indicated that HCT volunteer status was most strongly predicted by Honesty-Humility (OR: 162441.586, 95% CI: 19693.678, 1339885.277, d = 6.615) (Table 4). Openness to Experience was the next most strongly associated with volunteer group membership (OR: 10.089, 95% CI: 6.417, 15.862, d = 1.274). Emotionality (OR: 0.238, 95% CI: 0.080, 0.709, d = 0.791) and Conscientiousness (OR: 0.012, 95% CI: 0.002, 0.071, d = 2.438) were negatively predictive of volunteer status (i.e. more likely to predict control status). The other two dimensions were not statistically significant predictors.

**Table 4:** Odds of challenge volunteer membership by HEXACO dimension using logistic regression model, adjusted for gender, age, education, country of residence and income.

HEXACO Dimension	Wald statistic	p-value	Odds Ratio	95% CI for OR	Cohen’s d
***Honesty/Humility***	124.207	< 0.001	162441.586	19693.678, 1339885.277	6.615
***Emotionality***	6.644	0.010	0.238	0.080, 0.709	0.791
***eXtraversion***	0.917	0.338	0.810	0.527, 1.246	
***Agreeableness***	0.009	0.923	0.968	0.498, 1.882	
***Conscientiousness***	23.310	< 0.001	0.012	0.002, 0.071	2.438
***Openness to Experience***	100.240	< 0.001	10.089	6.417, 15.862	1.274

In addition, education level and income were both found to be significantly associated with volunteer group membership with a large effect size (Table 5). For example, study participants with an education level equivalent to a Bachelor’s degree had 264-fold increased odds of being a member of the volunteer group compared to those with less than high school equivalent education (OR: 264.752, p<0.001, d = 3.076). Participants with an annual income of greater than $200,000 had 4.5-fold odds of being a member of the volunteer group compared to those earning less than $25,000 annually. This model had a total Cox & Snell R^2^ = 0.439 (with HEXACO dimension covariates accounting for 16.4% of R^2^) and a -2 log-likelihood value of 2248.019, indicating that the addition of demographic covariates improved the fit of the model overall.

**Table 5:** Odds of challenge volunteer membership by gender, age, education, country of residence and income using logistic regression model, adjusted for HEXACO dimensions.

Covariate	Category	Wald	p-value	Odds Ratio	Cohen’s d
***Age***		203.985	< 0.001	0.935	0.037
***Country of Residence***	***US Resident***	65.325	< 0.001	0.021	2.130
***Gender***	***Female***	67.567	< 0.001	0.331	0.610
	***Self-Describe***	8.271	0.004	3.883	0.748
	***Prefer not to say***	1.182	0.227	0.570	
***Education***	***High School***	12.908	< 0.001	41.749	2.057
	***Associate***	17.209	< 0.001	79.712	2.414
	***Some college***	23.213	< 0.001	149.909	2.762
	***Bachelor’s***	29.026	< 0.001	264.752	3.076
	***Masters***	32.782	< 0.001	388.911	3.288
	***Doctoral***	39.235	< 0.001	862.968	3.727
	***Professional***	24.385	< 0.001	185.920	2.881
	***Trade/Technical***	20.810	< 0.001	132.826	2.695
***Income***	***$25k-$50k***	5.403	0.020	1.686	0.288
	***$50k-$100k***	25.936	< 0.001	3.091	0.622
	***$100k-$200k***	8.541	0.003	1.901	0.354
	***$200k +***	28.533	< 0.001	4.545	0.835
	***Prefer not to say***	44.834	< 0.001	13.426	1.432

### Risk sensitivity

We next compared risk behaviors and evaluations across the two groups. We predicted that volunteers would not, in general, exhibit more risk-taking behaviors or risk insensitivity relative to controls [54]. We compared groups on the six DOSPERT risk domains for each of the three components using an ANCOVA model (S7 Table), which included additional covariates for age, income, education level, gender, and US residency to control for the potential role of demographic differences between volunteers and controls. Results indicated that volunteers differed from controls in risk-taking attitudes in all domains except for financial investment. However, the volunteer group was not consistently the more risk-seeking group. Relative to controls, volunteers demonstrated greater risk-aversion in the domains of ethics (V: 1.46, C: 2.34, p<0.001, η^2^ = 0.136), financial-gambling scenarios (V: 1.40, C: 2.34, p<0.001,η^2^ = 0.107), and health and safety (V: 2.43, C: 2.94, p<0.001,η^2^ = 0.047), albeit at a lower effect size than the previous two. By contrast, volunteers were more risk-seeking than controls with respect to recreational activities and social behaviors (for example, challenging norms or authority). The effect size of risk-seeking was greatest within the social domain (V: 2.59, C: 2.14, p<0.001, η^2^ = 0.129).

We also identified significant differences between volunteers and controls on the *risk-perception* component of the DOSPERT across all domains, with the exception of the ethical domain. Volunteers perceived higher levels of risk than controls in the domain of financial-gambling, and perceived less risk than controls in the domains of financial investing, health and safety, recreational activities, and social behaviors. The effect sizes were mostly small or trivial (η^2^ <0.06), except for the perception of social behavior risk perception, (V: 1.62, C: 1.99, p < 0.001, η^2^ = 0.066).

Finally, with respect to the *perceived-benefits scale* of the DOSPERT, volunteers perceived risk-taking behaviors in the ethical (V: 1.87, C: 2.62, p < 0.001, η^2^ = 0.097), financial-gambling (V: 1.96, C: 2.82, p < 0.001, η^2^ = 0.075), and health and safety domains (V: 1.62, C: 2.35, p < 0.001, η^2^ = 0.100) as significantly less beneficial than did controls (all medium effect sizes). Exceptions included the recreational (V: 2.50, C: 2.36, p = 0.005, η^2^ = 0.003) and social domains (V: 2.91, C: 2.63, p < 0.001, η^2^ = 0.025), which volunteers perceived as more beneficial than did controls (although differences had trivial and small effect sizes, respectively). See S3 File for Dospert Fitted Score Descriptive Statistics for risk-taking likelihood, risk perception and expected benefits.

Further analyses regarding risk-perception relating to COVID-19 and volunteering for HCT participation identified in the pre-registration plan were beyond the scope of this paper and will be discussed in forthcoming papers.

## Discussion

Together, these results indicate that the characteristics of individuals expressing interest in and intention to volunteer for COVID-19 HCTs do not substantiate concerns regarding vulnerability or undue influence. Interest in volunteerism was overwhelmingly associated with heightened altruistic motivation and behavior. Nearly all volunteers reported altruistic motivations for volunteering, and demonstrated high levels of prior engagement in other forms of altruism, including donating blood, donating money to charity, and registering as living marrow donors and deceased organ donors. Volunteers also scored higher in personality traits like Honesty-Humility that reflect high valuation of others relative to the self [36]. Together, these metrics suggest that those who express interest in participating in COVID-19 HCTs (the benefits of which primarily accrue to others) exhibit reliably altruistic motivations, preferences, and values consistent with the nature of these trials.

We did not find evidence that interest in COVID-19 HCT volunteerism is disproportionately associated with psychological or demographic factors that might raise ethical concerns. Comparing risk perceptions and behaviors between volunteers and controls, we found that group differences were generally small in magnitude and did not suggest that volunteers were generally insensitive to factors that compromise physical health or safety. Although volunteers indicated that they would be more likely than controls to take risks in social, recreational, and investment domains, they indicated being less likely to take risks in the health and safety domain. Group differences in ratings may reflect in part the different risk/benefit profiles that the two groups perceived for different categories of risk. Volunteers perceived slightly lower risks in the health and safety domain than controls (η^2^ = 0.003), but also perceived lower benefits to activities in that domain (η^2^ = 0.100).

We also found no evidence that interest in COVID-19 HCT volunteerism is associated with high levels of socioeconomic vulnerability that might make volunteers subject to exploitation. Still, we cannot rule out the possibility that, had COVID-19 HCTs happened at that time, financial compensation may nonetheless have attracted people seeking economic gain, which might be construed as coercion or undue inducement to participate (although genuine offers of financial compensation are not considered coercive among bioethicists, see Largent et al. [55] for a review of the debate on coercion and undue inducement). Our results suggest that such HCTs would likely have been able to attract participants with non-economic motives, as volunteers in our sample reported higher levels of income and education relative to population medians and relative to controls, and equivalent levels of health insurance as controls. Even if some volunteers were attracted to participating in HCTs by the financial compensation, it still seems better if study participation aligns with participants’ existing altruistic traits than for participants to be induced by compensation to engage in a study that is not consistent with their values. Even for a primarily economically motivated volunteer, therefore, it is ethically preferable if there is also altruistic motivation. The high median educational attainment of volunteers (over three-quarters of whom reported having a bachelor’s degree or higher) also matters, as it suggests that volunteers are relatively well-positioned to understand the information disclosed during the consent process [56].

Of note, the majority of volunteers were male and between the ages of 18 and 45. A high proportion (78.5%) identified as non-Hispanic white. These socio-demographic variables confer both risk factors for and protective factors against serious COVID-19 outcomes. It is generally accepted that HCTs should include only young and medically healthy volunteers [2, 57], but the role that other socio-demographic risk factors should play in volunteer enrollment is debated. Male biological sex confers clear risks of serious illness or death following COVID-19 infection, with males’ average case-fatality ratio being 1.7 times higher than females’, an effect thought to reflect sex-based differences in innate and adaptive immune responses [58, 59]. COVID-19 related fatalities and hospitalizations are dramatically elevated among participants who identify as Black, Latino, and Native American in the US, as well as those of Asian ancestry in the UK. These findings are likely due to structural inequities and socioeconomic factors affecting health [60]. Some advocates of COVID-19 HCTs have proposed including volunteers from diverse backgrounds to ensure adequate representation of demographic groups that have been hardest hit by the pandemic [61]. More than twenty percent of the over 38,000 volunteers recruited through 1Day Sooner come from underrepresented groups, suggesting that HCTs enrolling from this pool could include a diverse group of participants.

Notably, our sample of intended volunteers is in some ways similar to “typical” healthy volunteer research participants in clinical trials in that they are predominantly male. But they are markedly different from what has been observed in previous studies of phase I study participants [24, 62] in that the intended volunteers in our sample were less likely to belong to demographic or socioeconomic groups most vulnerable to exploitation, including ethnic minorities, low-income earners, or unemployed individuals. This suggests that existing concerns about healthy volunteers who participate in other types or research in different contexts, do not necessarily generalize to healthy volunteers who were willing to participate in a COVID-19 HCT early in the pandemic. One could speculate that the social salience of COVID-19 early in the pandemic and the resulting media attention given to vaccine trials, including the possibility of HCTs, raised awareness among a wider population than would normally know about opportunities to be a healthy volunteer in medical research. The extreme potential social value of HCTs as a way to speed up the development of the first COVID-19 vaccines could have spurred the altruistically motivated to seek out ways to contribute. If this is correct, then more media attention and more emphasis on the social benefits of other research using healthy volunteers might also help other research to recruit more altruistic volunteers, potentially reducing the prevalence of possibly exploitative recruitment of vulnerable participants.

Together, our findings are inconsistent with concerns expressed early in the pandemic that HCTs with the novel coronavirus would be “*prima facie* unethical” because they would be expected to follow a “pattern of exploitative recruitment” [11]. Whereas HCT recruitment could be viewed as inherently exploitative if it attracted volunteers who find participation “very attractive as a result of being in a socioeconomically disadvantaged position as a result of social injustice” [11] or whose volunteerism reflects “financial desperation” [63], our results indicate that such trials, in principle, tend to attract volunteers who are primarily motivated by altruism and do not on the whole exhibit any indicators of socioeconomic or psychological vulnerability to exploitation.

Our findings should be interpreted in light of certain limitations. First, the survey was conducted in a sample of early volunteers who signed up with 1Day Sooner in April and May of 2020, the earliest weeks of its creation. Volunteers sampled here comprise only a subset of the total number of people interested in participating in COVID-19 HCTs at that time, and so may not be representative of all volunteers, and those who have subsequently volunteered may be different. Further, we acknowledge that it is possible that the volunteers who completed our survey were on average more engaged, interested, or eager to volunteer than volunteers who did not complete the survey. It is possible that volunteers’ responses were influenced by social desirability bias. However, it is unclear why HCT volunteers would experience greater social desirability bias than would those in the control group. Also arguing against social desirability bias driving our results were the consistent group differences across multiple prior altruistic behaviors. Multiple HEXACO scales are construed as desirable traits (including extraversion, agreeableness, conscientiousness, and openness) but the Honesty-Humility scale was most strongly associated with HCT volunteer status. If volunteers were simply selecting more socially desirable answers, they would have also self-reported themselves to be more agreeable, conscientious, etc., which was largely not the case.

We also cannot know what proportion of intended volunteers would have actually consented to participate in a COVID-19 HCT and passed exclusionary screening, if such a trial had taken place at the time of the survey. It is possible that this subset would be small or non-representative of the volunteers characterized in our study, similar to observations that altruistic marrow donors represent only a fraction of those who initially volunteer to donate [64]. However, we cannot make assumptions regarding specific changes in the composition of volunteers. In addition, our sample of controls, while recruited to reflect national United States characteristics established by 2019 census data (including age, gender, education and income), are not truly representative of the United States population as a whole. We cannot rule out the possibility of volunteers having inaccurate perceptions of the risks of participating in a COVID-19 HCT: although we asked about respondents’ perceptions of associated risks, there was no scientific consensus at the time that we could use as a standard to make claims about their accuracy. Nor can we rule out, based on our data, the possibility that COVID-19 HCT volunteerism reflects unmeasured biases related to the perception of risks and benefits, such as optimism bias [10, 65]; the so-called preventative or therapeutic fallacy, which reflects a common assumption that any treatment offered by medical professionals must be potentially beneficial [66, 67]; or unrealistic beliefs about potential personal gains. To some degree, such concerns can be resolved through a robust informed consent process [25, 26, 68], which is broadly viewed as possible for COVID-19 HCTs [2, 57, 69–71]. If, as our findings suggest, volunteers are mostly prepared to take the personal risks associated with such studies to benefit the greater good, then, given the large number of volunteers to come forth in a short amount of time, we can expect that there will be a sufficient number of altruistic volunteers able to provide valid consent to make these trials both ethical and feasible.

Lastly, our findings cannot be extrapolated to all HCTs, not even to all COVID-19 HCTs regardless of context. It cannot be assumed that this same pool of intended volunteers, or volunteers with similar characteristics, would continue to be willing to participate in COVID-19 HCTs at the current, later stage of the pandemic. The survey reported here captures a particular, early-pandemic social and medical context. This context, and the state of knowledge about the virus and vaccines, have changed dramatically since then and will continue to change. For the same reason, our findings should not be interpreted as applying to volunteers for HCTs in non-pandemic or non-epidemic contexts.

## Conclusions

Self-interest is sometimes incorrectly assumed to be the central or sole value driving human decisions [72, 73], which may contribute to pervasive concerns that volunteerism for risky and primarily other-benefiting biomedical procedures inevitably, or almost inevitably, indicates undue inducement or failures of informed consent. However, people vary widely in their selfish versus altruistic preferences and values [36, 74]. Those who volunteer for biomedical procedures that confer net personal risks and burdens without direct benefits (like kidney and marrow donations) place unusually high value on others’ welfare relative to their own [64, 75]. Such donations are now broadly accepted as ethical despite their risks and absence of direct benefits to volunteers because they are consistent with donors’ values and preferences. In finding that COVID-19 HCTs can attract volunteers whose altruistic preferences and values align with the nature of these trials (and who are not unusually vulnerable to exploitation), the present report may allay some ethical concerns about the volunteers interested in participating in COVID-19 HCTs. Our findings also suggest that similarly altruistically motivated volunteers may come forward in the early stages of future pandemics, when the social value of HCTs is arguably highest. Therefore, in future early pandemics, the *prima facie* concerns about undue inducement or failure of consent, and common assumptions and worries about vulnerable healthy volunteers elsewhere in biomedical research, should not be assumed to apply to HCTs of an emerging pandemic pathogen.

## Supporting information

S1 File Motivation question EFA. (DOCX)

S2 File Discussion on large observed odds ratio values. (DOCX)

S3 File DOSPERT fitted score descriptive statistics. (DOCX)

S4 File Survey questions. (DOCX)

S1 Table HEXACO factor loadings. (DOCX)

S2 Table HEXACO factors for the 24-question HEXACO survey. (DOCX)

S3 Table DOSPERT factor loadings: Risk-taking likelihood. (DOCX)

S4 Table DOSPERT factor loadings: Risk perception. (DOCX)

S5 Table DOSPERT factor loadings: Perceived benefits. (DOCX)

S6 Table DOSPERT scale factors and corresponding questions in the survey. (DOCX)

S7 Table Comparisons of DOSPERT risk attitude component scores. (DOCX)
