# Supplementary material for: Characterizing altruistic motivation in potential volunteers for SARS-CoV-2 challenge trials
Source: PLoS One. 2022 Nov 2;17(11):e0275823. doi: 10.1371/journal.pone.0275823 (PMC9629635; doi:10.1371/journal.pone.0275823)
Supplement: S1 File — (DOCX) [file pone.0275823.s001.docx]

## **S1 Supplementary Methods**

**Motivation Question EFA**:

An exploratory factor analysis was performed on the responses of HCT volunteers to the following questions regarding their motivations for volunteering:

Question 2.6_1: I want to help others and potentially save lives

Question 2.6_2: I feel helpless and this is a way to do something positive

Question 2.6_3: I was curious about COVID-19

Question 2.6_4: I wanted to be part of a clinical trial

Question 2.6_5: I wanted to contribute to the progress of medicine

Question 2.6_6: I wanted to find out more about my own health

Question 2.6_7: I wanted to receive financial reimbursement for participating

Question 2.6_8: I wanted to be guaranteed access to critical care should I be infected with COVID-19

Question 2.6_9: I am likely to be infected with COVID-19 anyway

The EFA analysis gave a 3 factor solution (variance explained 60.2%), with results given below.

**Rotated Component Matrix (Varimax Rotation):**

| Question number | Factor 1 | Factor 2 | Factor 3 |
| --- | --- | --- | --- |
| Question 2.6_1 | -0.478 | 0.374 | 0.534 |
| Question 2.6_2 | 0.174 | 0.003 | 0.832 |
| Question 2.6_3 | 0.273 | 0.688 | 0.026 |
| Question 2.6_4 | 0.163 | 0.790 | -0.069 |
| Question 2.6_5 | -0.253 | 0.730 | 0.236 |
| Question 2.6_6 | 0.587 | 0.522 | -0.022 |
| Question 2.6_7 | 0.722 | 0.121 | -0.199 |
| Question 2.6_8 | 0.713 | 0.200 | 0.132 |
| Question 2.6_9 | 0.574 | -0.105 | 0.289 |

Resulting factors (with suggested titles from the author):

**Factor 1 (Personal Benefit):**

Question 2.6_6: I wanted to find out more about my own health

Question 2.6_7: I wanted to receive financial reimbursement for participating

Question 2.6_8: I wanted to be guaranteed access to critical care should I be infected with COVID-19

Question 2.6_9: I am likely to be infected with COVID-19 anyway

**Factor 2 (Curiosity/Science):**

Question 2.6_3: I was curious about COVID-19

Question 2.6_4: I wanted to be part of a clinical trial

Question 2.6_5: I wanted to contribute to the progress of medicine

**Factor 3 (Altruism):**

Question 2.6_1: I want to help others and potentially save lives

Question 2.6_2: I feel helpless and this is a way to do something positive

Note: Question 2.6_6 (I want to find out more about my own health) had a loading of 0.587 into Factor 1, but also had a loading of 0.522 into Factor 2, so it might belong in Factor 2.
