# Supplementary material for: Characterizing altruistic motivation in potential volunteers for SARS-CoV-2 challenge trials
Source: PLoS One. 2022 Nov 2;17(11):e0275823. doi: 10.1371/journal.pone.0275823 (PMC9629635; doi:10.1371/journal.pone.0275823)
Supplement: S2 File — (DOCX) [file pone.0275823.s002.docx]

**S2 Supplementary Methods**

**Discussion on large observed odds ratio values:**

The large observed OR values for the HEXACO measures may be due to lower model fit or the compressed range and standard deviation of the fitted scores (see Table A for descriptive statistics on the fitted HEXACO scores), which is particularly small for the Honesty/Humility factor (total range = 0.868, SD = 0.147).

**Table A: HEXACO Fitted Score Descriptive Statistics:**

| HEXACO Dimension | Mean (SD) | Minimum | Maximum |
| --- | --- | --- | --- |
| Honesty/Humility | 0.952 (0.147) | 0.313 | 1.181 |
| Emotionality | -0.807 (0.418) | -2.053 | 1.001 |
| eXtraversion | 3.522 (0.580) | 1.366 | 4.642 |
| Agreeableness | 2.215 (0.469) | 0.487 | 3.566 |
| Conscientiousness | 1.114 (0.223) | 0.238 | 1.656 |
| Openness to Experience | 2.006 (0.398) | 0.275 | 2.938 |
