# Supplementary material for: Characterizing altruistic motivation in potential volunteers for SARS-CoV-2 challenge trials
Source: PLoS One. 2022 Nov 2;17(11):e0275823. doi: 10.1371/journal.pone.0275823 (PMC9629635; doi:10.1371/journal.pone.0275823)
Supplement: S3 File — (DOCX) [file pone.0275823.s003.docx]

**S3 Supplementary Methods**

**DOSPERT Fitted Score Descriptive Statistics:**

**Table A: DOSPERT Fitted Score Descriptives: Risk-taking likelihood:**

| **DOSPERT Dimension** | **Mean (SD)** | **Minimum** | **Maximum** |
| --- | --- | --- | --- |
| Ethical | 1.748 (0.998) | 0.720 | 6.643 |
| Financial-Investment | 2.258 (0.820) | 0.697 | 4.879 |
| Financial-Gambling | 1.710 (1.176) | 0.898 | 6.708 |
| Health/Safety | 2.600 (1.001) | 1.062 | 7.434 |
| Recreational | 2.095 (0.920) | 0.633 | 4.431 |
| Social | 2.442 (0.529) | 0.460 | 3.563 |

**Table B: DOSPERT Fitted Score Descriptives: Risk Perception:**

| **DOSPERT Dimension** | **Mean (SD)** | **Minimum** | **Maximum** |
| --- | --- | --- | --- |
| Ethical | 4.927 (1.158) | 1.018 | 7.126 |
| Financial-Investment | 3.572 (0.840) | 0.774 | 5.418 |
| Financial-Gambling | 5.136 (1.446) | 0.890 | 6.728 |
| Health/Safety | 4.862 (1.089) | 1.026 | 7.182 |
| Recreational | 2.498 (0.686) | 0.574 | 4.018 |
| Social | 1.739 (0.585) | 0.549 | 3.843 |

**Table C: DOSPERT Fitted Score Descriptives: Expected Benefits:**

| **DOSPERT Dimension** | **Mean (SD)** | **Minimum** | **Maximum** |
| --- | --- | --- | --- |
| Ethical | 2.115 (1.010) | 0.976 | 6.832 |
| Financial-Investment | 2.668 (0.956) | 0.768 | 5.383 |
| Financial-Gambling | 2.239 (1.287) | 0.882 | 6.615 |
| Health/Safety | 1.856 (0.962) | 0.831 | 6.573 |
| Recreational | 2.456 (1.044) | 0.796 | 5.572 |
| Social | 2.818 (0.743) | 0.685 | 4.805 |
