# Supplementary material for: Characterizing altruistic motivation in potential volunteers for SARS-CoV-2 challenge trials
Source: PLoS One. 2022 Nov 2;17(11):e0275823. doi: 10.1371/journal.pone.0275823 (PMC9629635; doi:10.1371/journal.pone.0275823)
Supplement: S4 File — (DOCX) [file pone.0275823.s004.docx]

**S1 Survey Questions**

Below are the questions that were sent to each participant

Information that was provided to participants at the beginning of the survey:

This survey will not be used to assess eligibility or identify potential participants for any future human challenge trial studies. Your answers will remain anonymous, and have no bearing on your participation in further research, and will not influence your ability to participate in future research in any way.

A COVID-19 infection study is likely to span a couple of months, with at least several weeks spent isolated in a dedicated clinical facility. Initially, volunteers would be screened for infection and undergo antibody testing to ensure they have not been exposed to the virus. Subsequently, participants would receive either placebo or vaccine. The virus would be administered roughly 2-4 weeks later after participants have had time to mount an immune response to the vaccine, after which participants would be observed from the time of infection for another 2-4 weeks. If a volunteer tests positive or has symptoms of COVID-19, this period might be extended and they might be moved to additional clinical facilities as needed. Throughout the study period, participants would be tested extensively, with blood draws, stool samples and nasal swabs conducted on a daily or even more frequent basis. In addition to the risks of being infected with COVID-19, the experimental vaccine may have its own risks. We will not know in advance whether the experimental vaccine or controlled infection will enable volunteers to be protected against the virus in the future.

**Questionnaire**

**1.** **In what way did you first hear about the idea of a COVID-19 infection study?**

**2.** **What was your first reaction to the idea of a COVID-19 infection study?**

**3.** **When did you decide you wanted to enroll in a COVID-19 infection study?** *I.e. Is it a decision you made immediately or did you take some time to think about it?*

**Please indicate how important the following factors are in your decision to volunteer for a controlled COVID-19 infection study^[1]^:**

| **4.** **I want to help others and potentially save lives** | Strongly disagree | Somewhat disagree | Neither agree nor disagree | Somewhat agree | Strongly agree |
| --- | --- | --- | --- | --- | --- |
| **5.** **I feel helpless and this is a way to do something positive** | Strongly disagree | Somewhat disagree | Neither agree nor disagree | Somewhat agree | Strongly agree |
| **6.** **I was curious about COVID-19** | Strongly disagree | Somewhat disagree | Neither agree nor disagree | Somewhat agree | Strongly agree |
| **7.** **I wanted to be part of a clinical trial** | Strongly disagree | Somewhat disagree | Neither agree nor disagree | Somewhat agree | Strongly agree |
| **8.** **I wanted to contribute to the progress of medicine** | Strongly disagree | Somewhat disagree | Neither agree nor disagree | Somewhat agree | Strongly agree |
| **9.** **I wanted to find out more about my own health** | Strongly disagree | Somewhat disagree | Neither agree nor disagree | Somewhat agree | Strongly agree |
| **10.** **I wanted to receive financial reimbursement for participating** | Strongly disagree | Somewhat disagree | Neither agree nor disagree | Somewhat agree | Strongly agree |
| **11.** **I wanted to be guaranteed access to critical care should I be infected with COVID-19** | Strongly disagree | Somewhat disagree | Neither agree nor disagree | Somewhat agree | Strongly agree |
| **12.** **I am likely to be infected with COVID-19 anyway** | Strongly disagree | Somewhat disagree | Neither agree nor disagree | Somewhat agree | Strongly agree |

**13.** **Are there any other factors that influenced your decision to volunteer for a controlled COVID-19 infection study?**

**14.** **What are the three most important factors in your decision to participate?** *Please select three options*

❏ I want to help others and potentially save lives

❏ I feel helpless and this is a way to do something positive

❏ I was curious about COVID-19

❏ I wanted to be part of a clinical trial

❏ I wanted to contribute to the progress of medicine

❏ I wanted to find out more about my own health

❏ I want to receive the financial reimbursement for participating

❏ I wanted to be guaranteed access to critical care should I be infected with COVID-19

❏ I am likely to be infected with COVID-19 anyway

❏ Other factor not mentioned (please specify): ___________________________________________________

**15.** **Please indicate the extent to which you agree with the following statement: COVID-19 is of similar severity as influenza (the flu)**

| 1  Strongly disagree | 2  Disagree | 3  Somewhat disagree | 4  Undecided | 5  Somewhat agree | 6  Agree | 7  Strongly agree |
| --- | --- | --- | --- | --- | --- | --- |

**16.** **What do you think are the main risks and burdens of being in a controlled COVID-19 infection study?**

**a.** **Which of these risks and burdens concern you the most?**

**b.** **Which concern you the least?**

**17.** **What do you think are the main benefits of being in a controlled COVID-19 infection study?**

**18.** **How risky do you think a COVID-19 challenge study is overall, on a scale of 1 to 10?** This refers to the risk to your own health from participating — not a risk to others, and not including how likely you are to be infected with COVID-19 outside of a trial.

| 1  Not risky at all | 2 | 3 | 4 | 5  Moderately risky | 6 | 7 | 8 | 9 | 10  Extremely risky |
| --- | --- | --- | --- | --- | --- | --- | --- | --- | --- |

**19.** **What is your perception of the risk of developing conditions during a COVID-19 challenge trial that are severe enough to require critical care such as a ventilator?**

● 1 in 100,000 (0.001%)

● 1 in 50,000 (0.002%)

● 1 in 10,000 (0.01%)

● 1 in 5,000 (0.02%)

● 1 in 1,000 (0.1%)

● 1 in 100 (1%)

● 1 in 10 (10%)

● 1 in 5 (20%)

**20.** **How much care do you predict you would receive from the researchers if you were to become sick?**

| 1  None at all | 2  A little | 3  A moderate amount | 4  A lot | 5  A great deal |
| --- | --- | --- | --- | --- |

**21.** **How much care do you think you have the right to receive from the researchers if you were to become sick?**

| 1  None at all | 2  A little | 3  A moderate amount | 4  A lot | 5  A great deal |
| --- | --- | --- | --- | --- |

**22.** **How would you rate your risk of dying should you become infected with COVID-19 during the course of a challenge trial, if you were to participate?**

| 1  There is no risk to me | 2 | 3 | 4  There is a moderate risk to me | 5 | 6 | 7  The risk to me is extremely high |
| --- | --- | --- | --- | --- | --- | --- |

**23.** **What is your perception of your risk of dying should you become infected with COVID-19 during a challenge trial, if you were to participate?**

● 1 in 100,000 (0.001%)

● 1 in 50,000 (0.002%)

● 1 in 10,000 (0.01%)

● 1 in 5,000 (0.02%)

● 1 in 1,000 (0.1%)

● 1 in 100 (1%)

● 1 in 10 (10%)

● 1 in 5 (20%)

● 1 in 3 (33.3%)

**24.** **Compared to your expected risk in participating in a COVID-19 challenge trial, to what extent would you be willing to accept greater risk as a participant if you believed the benefits to society would be significant?**

| 1  I would not accept any additional risk | 2 | 3 | 4  I would accept a moderately greater level of risk | 5 | 6 | 7  I would accept a seriously greater level of risk |
| --- | --- | --- | --- | --- | --- | --- |

**25.** **What is the highest risk of death that you would accept to participate in a COVID-19 challenge trial if you believed such a trial was likely to confer significant benefits to society?**

● 1 in 100,000 (0.001%)

● 1 in 50,000 (0.002%)

● 1 in 10,000 (0.01%)

● 1 in 5,000 (0.02%)

● 1 in 1,000 (0.1%)

● 1 in 100 (1%)

● 1 in 10 (10%)

● 1 in 5 (20%)

● 1 in 3 (33.3%)

**26.** **How would you rate your risk of becoming infected with COVID-19 during the course of your everyday life in the next six months?**

| 1  There is no risk to me | 2 | 3 | 4  There is a moderate risk to me | 5 | 6 | 7  The risk to me is extremely high |
| --- | --- | --- | --- | --- | --- | --- |

**27.** **What is your perception of your risk of becoming infected with COVID-19 during the course of your everyday life in the next six months?**

● 1 in 100,000 (0.001%)

● 1 in 50,000 (0.002%)

● 1 in 10,000 (0.01%)

● 1 in 5,000 (0.02%)

● 1 in 1,000 (0.1%)

● 1 in 100 (1%)

● 1 in 10 (10%)

● 1 in 5 (20%)

● 1 in 3 (33%)

● 1 in 2 (50%)

● 3 in 4 (75%)

● 9 in 10 (90%)

● Almost certainly (95% or greater)

**28.** **How would you describe your risk of not being put on a ventilator or receiving other scarce treatments (such as dialysis machines) that you need to survive in your current living area because of the COVID-19 surge in demand for health services?**

| 1  There is no risk to me | 2 | 3 | 4  There is a moderate risk to me | 5 | 6 | 7  The risk to me is extremely high |
| --- | --- | --- | --- | --- | --- | --- |

**29.** **What is your perception of your risk of not being put on a ventilator or receiving other scarce treatments (such as dialysis machines) that you need to survive in your current living area because of the COVID-19 surge in demand for health services?**

● 1 in 100,000 (0.001%)

● 1 in 50,000 (0.002%)

● 1 in 10,000 (0.01%)

● 1 in 5,000 (0.02%)

● 1 in 1,000 (0.1%)

● 1 in 100 (1%)

● 1 in 10 (10%)

● 1 in 5 (20%)

● 1 in 3 (33%)

● 1 in 2 (50%)

● 3 in 4 (75%)

● 9 in 10 (90%)

● Almost certainly (95% or greater)

**30.** **How would you rate your risk of dying from COVID-19 during the course of your everyday life in the next six months?**

| 1  There is no risk to me | 2 | 3 | 4  There is a moderate risk to me | 5 | 6 | 7  The risk to me is extremely high |
| --- | --- | --- | --- | --- | --- | --- |

**31.** **What is your perception of your risk of dying from COVID-19 during the course of your everyday life in the next six months?**

● 1 in 100,000 (0.001%)

● 1 in 50,000 (0.002%)

● 1 in 10,000 (0.01%)

● 1 in 5,000 (0.02%)

● 1 in 1,000 (0.1%)

● 1 in 100 (1%)

● 1 in 10 (10%)

● 1 in 5 (20%)

**32.** **How many infections of COVID-19 do you think will have occurred worldwide, before 2021?**

● Between 2 million and 5 million infections

● Between 5 million and 20 million infections

● Between 20 million and 25 million infections

● Between 25 million and 50 million infections

● Between 50 million and 100 million infections

● Between 100 million and 250 million infections

● Between 250 million and 500 million infections

● Between 500 million and 750 million infections

● More than 750 million infections

**33.** **How many people do you think will die as a direct result of COVID-19 before 2021?**

● Under 20,000 deaths

● Between 20,000 and 50,000 deaths

● Between 50,000 and 100,000 deaths

● Between 100,000 and 250,000 deaths

● Between 250,000 and 500,000 deaths

● Between 500,000 and 750,000 deaths

● Between 750,000 and 1 million deaths

● Between 1 million and 2 million deaths

● Between 2 million and 5 million deaths

● Between 5 million and 10 million deaths

● More than 10 million deaths

**34.** **How many people do you think will die as a indirect result of COVID-19 before 2021?** *This might include people who die of conditions that clinicians must neglect in order to prioritize COVID-19, halted economic development, ensuing famine, etc.*

● Under 20,000 deaths

● Between 20,000 and 50,000 deaths

● Between 50,000 and 100,000 deaths

● Between 100,000 and 250,000 deaths

● Between 250,000 and 500,000 deaths

● Between 500,000 and 750,000 deaths

● Between 750,000 and 1 million deaths

● Between 1 million and 2 million deaths

● Between 2 million and 5 million deaths

● Between 5 million and 10 million deaths

● More than 10 million deaths

**35.** **How likely do you think it is that your participation in a COVID-19 human challenge trial leads to accelerated vaccine development?**

| 1  Extremely unlikely | 2  Fairly unlikely | 3  Somewhat unlikely | 4  Neutral | 5  Somewhat likely | 6  Fairly likely | 7  Extremely  likely |
| --- | --- | --- | --- | --- | --- | --- |

**36.** **How likely do you think it is that your participation in a COVID-19 human challenge trial leads to saving people’s lives?**

| 1  Extremely unlikely | 2  Fairly unlikely | 3  Somewhat unlikely | 4  Neutral | 5  Somewhat likely | 6  Fairly likely | 7  Extremely likely |
| --- | --- | --- | --- | --- | --- | --- |

**37.** **Did you talk to anyone about whether you should join a potential human challenge study?**

● Yes

● No

● Prefer not to say

**a.** **If yes, who did you talk to?**

**b.** **What did they think about it?**

**Please indicate whether you believe the following statements are true or false.** This section does not require any additional outside research, and is simply designed to capture your current understanding of how a human challenge trial may work.

**38.** Participants in a human challenge trial would be exposed to the novel coronavirus (COVID-19):

● True

● False

**39.** Participants in a human challenge trial will be isolated from their household and general public in a specialized health facility

● True

● False

**40.** Participants in a human challenge trial might infect their family members with COVID-19

● True

● False

**41.** Participants are eligible for a human challenge trial if they have already tested positive for COVID-19

● True

● False

**42.** All participants will receive the candidate COVID-19 vaccine

● True

● False

**43.** **Have you ever participated in research studies before?**

| Never | Once | More than once | Often | Very Often |
| --- | --- | --- | --- | --- |

**44.** **If you have participated in research studies before, tell us more about that experience.**

**a.** What was the purpose of the study?

**b.** Was your experience mostly positive or mostly negative?

**c.** How would this study be different from previous studies you have participated in?

**45.** **Have you ever donated blood before?**

| Never | Once | More than once | Often | Very Often |
| --- | --- | --- | --- | --- |

**46.** **Have you ever donated a significant amount of money to charity?**

● Yes, I have occasionally

● Yes, I donate annually

● No, I have never donated to charity

**47.** **Are you a registered bone marrow donor?**

● Yes

● No

● Unsure/I don’t know

● Prefer not to say

**48.** **Are you a registered deceased organ donor?**

● Yes

● No

● Unsure/I don’t know

● Prefer not to say

**49.** **Have you ever been a living kidney or liver donor?**

● Yes, I am a kidney donor

● Yes, I am a liver donor

● No

● Prefer not to say

**50.** **If a COVID-19 human challenge trial moves forward and you are asked to participate, what is the likelihood you agree to participate?**

| 1  Extremely unlikely | 2  Fairly unlikely | 3  Somewhat unlikely | 4  Neutral | 5  Somewhat likely | 6  Fairly likely | 7  Extremely likely |
| --- | --- | --- | --- | --- | --- | --- |

**51.** **If an effective treatment for COVID-19 is discovered, and a COVID-19 human challenge trial moves forward, what is the likelihood you agree to participate?**

| 1  Extremely unlikely | 2  Fairly unlikely | 3  Somewhat unlikely | 4  Neutral | 5  Somewhat likely | 6  Fairly likely | 7  Extremely likely |
| --- | --- | --- | --- | --- | --- | --- |

**52.** **If a COVID-19 human challenge trial moves forward and you are asked to participate, what would make you NOT want to enroll in such a study?**

**53.** **How likely do you think it is that some people participating in the study might not tell the researchers things that could disqualify them from participating?** *For example, not fully revealing drug or alcohol consumption, or personal history that may exclude them from participating otherwise.*

| 1  Extremely unlikely | 2  Fairly unlikely | 3  Somewhat unlikely | 4  Neutral | 5  Somewhat  likely | 6  Fairly likely | 7  Extremely  likely |
| --- | --- | --- | --- | --- | --- | --- |

**54.** **How do you think other people would perceive your enrollment in a COVID-19 challenge trial?**

| 1  Very negatively | 2  Fairly negatively | 3  Somewhat negatively | 4  Neutral | 5  Somewhat positively | 6  Fairly positively | 7  Very positively |
| --- | --- | --- | --- | --- | --- | --- |

**55.** **Where others (e.g., their parents or colleagues) may be upset about your enrollment, what would be helpful, if anything, for us or the trial team to do to pre-empt or address these reactions?** *For example, providing you with information about the study to share with others?*

**56.** **Please indicate whether any of your personal connections have been infected with COVID-19.**

*A severe case is defined here as requiring hospitalization or admission to an intensive care unit (ICU).*

| **Personal Connection** | **COVID-19 Status** |
| --- | --- |
| Partner/Spouse | N/A Mild Severe Fatal |
| Parent | N/A Mild Severe Fatal |
| Child | N/A Mild Severe Fatal |
| Sibling | N/A Mild Severe Fatal |
| Close friend | N/A Mild Severe Fatal |
| Work colleague | N/A Mild Severe Fatal |
| Housemate | N/A Mild Severe Fatal |
| Remote friend | N/A Mild Severe Fatal |
| Extended family | N/A Mild Severe Fatal |
| Classmate | N/A Mild Severe Fatal |
| Neighbor | N/A Mild Severe Fatal |
| Other: | N/A Mild Severe Fatal |

**57.** **Are any of your personal connections considered at-risk of becoming infected with COVID-19 or high-risk?** *At-risk might include healthcare workers, other front-line workers or people residing in areas heavily burdened by COVID-19.*

*High-risk might include people who are 70 or older, are pregnant, or have an underlying condition that increases their risk of COVID-19 complications.*

● Yes

● No

● Unsure/I don’t know

● Prefer not to say

**a.** If yes, please specify below. Complete for all that apply:

| **Personal Connection** | **COVID-19 Risk Designation** | **Lives in Household** |
| --- | --- | --- |
| Partner/Spouse | At-risk High- risk | Yes No |
| Parent | At-risk High- risk | Yes No |
|  |  |  |
| Child | At-risk High- risk | Yes No |
| Sibling | At-risk High- risk | Yes No |
| Close friend | At-risk High- risk | Yes No |
| Work colleague | At-risk High- risk | Yes No |
| Housemate | At-risk High- risk | Yes No |
| Remote friend | At-risk High- risk | Yes No |
| Extended family | At-risk High- risk | Yes No |
| Classmate | At-risk High- risk | Yes No |
| Neighbor | At-risk High- risk | Yes No |
| Other: | At-risk High- risk | Yes No |

**Please indicate the extent to which you agree with the following statements^[2]^:**

**58.** I have conﬁdence in doctors and medical professionals

| 1  Strongly disagree | 2  Disagree | 3  Somewhat disagree | 4  Undecided | 5  Somewhat agree | 6  Agree | 7  Strongly agree |
| --- | --- | --- | --- | --- | --- | --- |

**59.** I trust doctors’ judgement about my medical care

| 1  Strongly disagree | 2  Disagree | 3  Somewhat disagree | 4  Undecided | 5  Somewhat agree | 6  Agree | 7  Strongly agree |
| --- | --- | --- | --- | --- | --- | --- |

**60.** I have confidence in scientific researchers

| 1  Strongly disagree | 2  Disagree | 3  Somewhat disagree | 4  Undecided | 5  Somewhat agree | 6  Agree | 7  Strongly agree |
| --- | --- | --- | --- | --- | --- | --- |

***B. Demographic Information***

| **61.** **City** |  |
| --- | --- |
| **62.** **State** |  |
| **63.** **Post (ZIP) Code** |  |
| **64.** **Country** |  |
| **65.** **Year of Birth** |  |
| **66.** **Height** | cm OR ft inches |
| **67.** **Weight** | kg OR pounds |
| **68.** **Gender** | Male  Female  Prefer to self describe as ____________ (non-binary, gender-fluid, agender, please specify)  Prefer not to say |
| **69.** **What is your marital status?** | Single (never married)  Married, or in a domestic partnership  Widowed  Divorced  Separated |

**70.** **Please specify your ethnicity (select all that apply):**

● African

● Asian (East Asian)

● Asian (South Asian)

● Caucasian (Non-Hispanic)

● Carribean

● Latino or Hispanic

● Native American

● Native Hawaiian or Pacific Islander

● Other/Unknown

● Prefer not to say

**71.** **What is the highest degree or level of school you have completed?**

*If currently enrolled, the highest degree received.*

● Less than high school equivalent

● High school graduate, diploma or the equivalent (for example: GED)

● Some college credit, no degree

● Trade/technical/vocational training

● Associate degree

● Bachelor’s degree

● Master’s degree

● Professional degree

● Doctoral degree

**72.** **What is your work status? (select all that apply)**

● Employed

● Self-employed/Freelance

● Interning

● Part-time

● Unemployed- Looking for work

● Unemployed – Not looking for work

● Homemaker

● Studying

● Military/Armed Forces

● Retired

● Not able to work

● Other

**73.** **Are you currently working from home?**

● Yes, full-time

● Yes, part-time (and part-time from my workplace)

● No

**74.** **Are you classified as an essential worker by your government?** *(Essential sectors could include health and social care, education and childcare, key public services—justice system, mortuaries, journalists —local and national government, those involves in food or other necessary goods production, distribution or sale and delivery, public safety and national security, transport, or utilities, communication and financial services)*

● Yes

● No

● I’m not sure

**75.** **What is your annual household income?**

● Less than $25,000 USD

● $25,000 - $50,000 USD

● $50,000 - $100,000 USD

● $100,000 - $200,000 USD

● More than $200,000 USD

● Prefer not to say

**76.** **Do you currently have health insurance?**

● Yes

● No

● I’m not sure

**a.** **If yes, who pays for your health insurance? (Check all that apply)**

● Government

● Current employer

● Former employer

● Self-funded

● Other: ________

**77.** **Do you have children? If yes, how many?**

● Yes, I have ___ children

● No

**78.** **Do you have dependents? If yes, how many?** *Dependents refer to people you financially support and would not include a spouse that generates their own income but may include children or elderly parents, etc.*

● Yes, I have ___ dependents (include the children from your answer above if they are dependent on you)

● No

**79.** **How many other people do you share your household with?**

| 0 | 1 | 2 | 3 | 4 | 5 | 6 | 7 | 8+ |
| --- | --- | --- | --- | --- | --- | --- | --- | --- |

**80.** **How many rooms are there in your household?** *This includes kitchens, living rooms, bedrooms, bathrooms, etc.*

| 0 | 1 | 2 | 3 | 4 | 5 | 6 | 7 | 8+ |
| --- | --- | --- | --- | --- | --- | --- | --- | --- |

**81.** **Do you have any of the following medical issues? (Select all that apply)**

● Asthma

● Blood disorders (such as sickle cell disease or taking blood thinners (*anticoagulants*))

● Cancer

● Chronic kidney disease

● Chronic liver disease

● Compromised immune system

● Currently pregnant or a recent pregnancy in the last two weeks

● Endocrine disorders, including diabetes

● Heart disease

● High blood pressure

● Lung disease, including asthma

● Metabolic disorders

● Neurological and neurologic and neurodevelopment conditions

**82.** **Do you currently smoke tobacco?**

● Yes, daily

● Yes, but not daily

● No, but I used to smoke daily

● No, but I used to smoke less than daily

● No, I have never smoked tobacco

● I’d rather not say

**83.** **Do you currently smoke marijuana?**

● Yes, daily

● Yes, but not daily

● No, but I used to smoke daily

● No, but I used to smoke less than daily

● No, I have never smoked marijuana

● I’d rather not say

**84.** **Do you currently use vaping products?**

● Yes, daily

● Yes, but not daily

● No, but I used to use them daily

● No, but I used to use them less than daily

● No, I have never used vaping products

● I’d rather not say

#

# **Frameworks/Tools:**

**The HEXACO Personality Index (10-15 minutes)**

● A measure of the six major dimensions of personality

● <https://hexaco.org/>

**DOSPERT Scale (15-20 minutes)**

● Assesses both conventional risk attitudes (defined as the reported level of risk taking) and perceived-risk attitudes (defined as the willingness to engage in a risky activity as a function of its perceived riskiness) in five commonly encountered content domains, i.e., ethical, financial (further decomposed into gambling and investment), health/safety, social, and recreational decisions.

● Weber, E. U., Blais, A.-R., & Betz, N. (2002). A domain-specific risk-attitude scale: Measuring risk perceptions and risk behaviors. Journal of Behavioral Decision Making, 15, 263-290.

● Blais, A.-R., & Weber, E. U. (2006). A domain-specific risk-taking (DOSPERT) scale for adult populations. *Judgment and Decision Making*, *1*(1).

^[1]^Oguti, B., Gibani, M., Darlow, C., Waddington, C. S., Jin, C., Plested, E., Campbell, D., Jones, C., Darton, T. C., & Pollard, A. J. (2019). [Factors influencing participation in controlled human infection models](https://doi.org/10.12688/wellcomeopenres.15469.1): a pooled analysis from six enteric fever studies. Wellcome Open Research, 4, 153.

^[2]^ Partially adapted from Kelly, J. J., Njuki, F., Lane, P. L., & McKinley, R. K. (2005). [Design of a Questionnaire to Measure Trust in an Emergency Department.](https://doi.org/10.1111/j.1553-2712.2005.tb00853.x) *Academic Emergency Medicine*, *12*(2), 147–151.
