# Supplementary material for: Characterizing altruistic motivation in potential volunteers for SARS-CoV-2 challenge trials
Source: PLoS One. 2022 Nov 2;17(11):e0275823. doi: 10.1371/journal.pone.0275823 (PMC9629635; doi:10.1371/journal.pone.0275823)
Supplement: S1 Table — (DOCX) [file pone.0275823.s005.docx]

**S1 Table. HEXACO Factor Loadings**

| **Factor Score Weights - HEXACO** | |  |  |  |  |  |
| --- | --- | --- | --- | --- | --- | --- |
|  | Honesty  Humility | Emotionality | eXtraversion | Agreeableness | Conscientiousness | OpenExperience |
| HEXACO19 | -0.003 | -0.031 | 0.016 | 0.011 | 0.000 | 0.152 |
| HEXACO13 | -0.004 | -0.045 | 0.023 | 0.016 | 0.001 | 0.218 |
| HEXACO7R | -0.001 | -0.015 | 0.008 | 0.006 | 0.000 | 0.075 |
| HEXACO1 | -0.002 | -0.022 | 0.011 | 0.008 | 0.000 | 0.106 |
| HEXACO20R | 0.010 | -0.056 | 0.019 | 0.019 | 0.065 | 0.001 |
| HEXACO14 | 0.007 | -0.037 | 0.013 | 0.012 | 0.043 | 0.001 |
| HEXACO8R | 0.013 | -0.072 | 0.024 | 0.024 | 0.083 | 0.001 |
| HEXACO2 | 0.004 | -0.023 | 0.008 | 0.008 | 0.027 | 0.000 |
| HEXACO21 | 0.004 | -0.034 | 0.025 | 0.120 | 0.006 | 0.006 |
| HEXACO15 | -0.001 | 0.010 | -0.007 | -0.034 | -0.002 | -0.002 |
| HEXACO9R | 0.003 | -0.026 | 0.019 | 0.090 | 0.004 | 0.005 |
| HEXACO3R | 0.006 | -0.056 | 0.042 | 0.197 | 0.009 | 0.010 |
| HEXACO22R | 0.012 | -0.037 | 0.222 | 0.062 | 0.014 | 0.022 |
| HEXACO16 | 0.005 | -0.014 | 0.086 | 0.024 | 0.005 | 0.008 |
| HEXACO10 | 0.003 | -0.009 | 0.056 | 0.015 | 0.003 | 0.005 |
| HEXACO4R | 0.015 | -0.043 | 0.260 | 0.072 | 0.016 | 0.025 |
| HEXACO23 | 0.000 | 0.029 | -0.006 | -0.013 | -0.006 | -0.006 |
| HEXACO17R | 0.002 | 0.186 | -0.035 | -0.080 | -0.038 | -0.039 |
| HEXACO11R | 0.001 | 0.063 | -0.012 | -0.027 | -0.013 | -0.013 |
| HEXACO5 | 0.001 | 0.097 | -0.018 | -0.042 | -0.020 | -0.021 |
| HEXACO24R | 0.059 | 0.010 | 0.047 | 0.033 | 0.027 | -0.015 |
| HEXACO18R | 0.034 | 0.005 | 0.027 | 0.018 | 0.015 | -0.008 |
| HEXACO12R | 0.061 | 0.010 | 0.048 | 0.033 | 0.028 | -0.015 |
| HEXACO6 | 0.007 | 0.001 | 0.006 | 0.004 | 0.003 | -0.002 |

**S1 Table:** CFA Factor loadings for the HEXACO survey. HEXACO question numbers are given in the first column (with an R denoting those questions that are reverse coded) and the factor loadings given under the six HEXACO dimension headings.
