# Supplementary material for: Characterizing altruistic motivation in potential volunteers for SARS-CoV-2 challenge trials
Source: PLoS One. 2022 Nov 2;17(11):e0275823. doi: 10.1371/journal.pone.0275823 (PMC9629635; doi:10.1371/journal.pone.0275823)
Supplement: S2 Table — (DOCX) [file pone.0275823.s006.docx]

**S2 Table. HEXACO Factors for the 24-question HEXACO Survey**

| **HEXACO Factors** |
| --- |
| Honesty/Humility: Questions 6, 12R, 18R, 24R |
| Emotionality: Questions 5, 11R, 17R, 23 |
| eXtraversion: Questions 4R, 10, 16, 22R |
| Agreeableness: Questions 3R, 9R, 15, 21 |
| Conscientiousness: Questions 2, 8R, 14, 20R |
| Openness to Experience: Questions 1, 7R, 13, 19 |

**S2 Table:** Details of the questions on the 24-question HEXACO survey corresponding to the original six HEXACO factors. Question numbers with an R denote questions that were reverse-coded for analysis.
