# Supplementary material for: Characterizing altruistic motivation in potential volunteers for SARS-CoV-2 challenge trials
Source: PLoS One. 2022 Nov 2;17(11):e0275823. doi: 10.1371/journal.pone.0275823 (PMC9629635; doi:10.1371/journal.pone.0275823)
Supplement: S3 Table — (DOCX) [file pone.0275823.s007.docx]

**S3 Table. DOSPERT Factor Loadings: Risk-Taking Likelihood**

| Factor Score Weights - DOSPERT Risk-Taking Likelihood | | |  |  |  |  |
| --- | --- | --- | --- | --- | --- | --- |
|  | FinanceInvest | FinanceGamble | HealthSafety | Recreation | Social | Ethical |
| Q7.4 | 0.070 | 0.005 | 0.004 | 0.005 | 0.005 | 0.002 |
| Q7.12 | 0.164 | 0.011 | 0.008 | 0.011 | 0.012 | 0.005 |
| Q7.18 | 0.208 | 0.014 | 0.011 | 0.014 | 0.015 | 0.006 |
| Q7.3 | 0.023 | 0.250 | 0.022 | 0.001 | -0.005 | 0.041 |
| Q7.8 | 0.026 | 0.279 | 0.025 | 0.001 | -0.006 | 0.046 |
| Q7.14 | 0.025 | 0.267 | 0.024 | 0.001 | -0.005 | 0.044 |
| Q7.5 | 0.004 | 0.005 | 0.070 | 0.007 | 0.005 | 0.028 |
| Q7.15 | 0.003 | 0.004 | 0.065 | 0.007 | 0.004 | 0.026 |
| Q7.17 | 0.006 | 0.008 | 0.116 | 0.012 | 0.008 | 0.046 |
| Q7.20 | 0.006 | 0.007 | 0.114 | 0.012 | 0.008 | 0.045 |
| Q7.23 | 0.002 | 0.003 | 0.039 | 0.004 | 0.003 | 0.015 |
| Q7.26 | 0.003 | 0.003 | 0.050 | 0.005 | 0.003 | 0.020 |
| Q7.2 | 0.004 | 0.000 | 0.006 | 0.033 | 0.003 | 0.000 |
| Q7.11 | 0.007 | 0.000 | 0.010 | 0.060 | 0.005 | 0.000 |
| Q7.13 | 0.008 | 0.000 | 0.013 | 0.076 | 0.006 | 0.000 |
| Q7.19 | 0.014 | 0.001 | 0.022 | 0.130 | 0.010 | 0.000 |
| Q7.24 | 0.014 | 0.001 | 0.022 | 0.130 | 0.010 | 0.000 |
| Q7.25 | 0.008 | 0.000 | 0.013 | 0.078 | 0.006 | 0.000 |
| Q7.1 | 0.012 | -0.002 | 0.011 | 0.008 | 0.076 | -0.007 |
| Q7.7 | 0.011 | -0.002 | 0.010 | 0.007 | 0.071 | -0.007 |
| Q7.21 | 0.010 | -0.002 | 0.010 | 0.007 | 0.068 | -0.007 |
| Q7.22 | 0.015 | -0.003 | 0.014 | 0.010 | 0.097 | -0.009 |
| Q7.27 | 0.009 | -0.002 | 0.008 | 0.006 | 0.057 | -0.006 |
| Q7.28 | 0.010 | -0.002 | 0.010 | 0.007 | 0.068 | -0.007 |
| Q7.30 | 0.003 | 0.011 | 0.036 | 0.000 | -0.004 | 0.066 |
| Q7.29 | 0.002 | 0.009 | 0.028 | 0.000 | -0.003 | 0.051 |
| Q7.16 | 0.003 | 0.014 | 0.045 | 0.000 | -0.005 | 0.082 |
| Q7.10 | 0.010 | 0.044 | 0.143 | 0.001 | -0.016 | 0.262 |
| Q7.9 | 0.003 | 0.015 | 0.049 | 0.000 | -0.005 | 0.089 |
| Q7.6 | 0.004 | 0.020 | 0.064 | 0.000 | -0.007 | 0.117 |

**S3 Table:** CFA Factor loadings for the DOSPERT survey. DOSPERT question numbers are given in the first column (Q7 is risk-taking likelihood) and the factor loadings given under the six DOSPERT dimension headings.
