# Supplementary material for: Characterizing altruistic motivation in potential volunteers for SARS-CoV-2 challenge trials
Source: PLoS One. 2022 Nov 2;17(11):e0275823. doi: 10.1371/journal.pone.0275823 (PMC9629635; doi:10.1371/journal.pone.0275823)
Supplement: S4 Table — (DOCX) [file pone.0275823.s008.docx]

**S4 Table. DOSPERT Factor loadings: Risk Perception**

| Factor Score Weights - DOSPERT Risk Perception | | |  |  |  |  |
| --- | --- | --- | --- | --- | --- | --- |
|  | FinanceInvest | FinanceGamble | HealthSafety | Recreation | Social | Ethical |
| Q8.4 | 0.084 | 0.010 | 0.003 | 0.005 | 0.004 | 0.001 |
| Q8.12 | 0.200 | 0.023 | 0.008 | 0.011 | 0.009 | 0.003 |
| Q8.18 | 0.223 | 0.026 | 0.008 | 0.012 | 0.010 | 0.004 |
| Q8.3 | 0.027 | 0.212 | 0.014 | 0.000 | -0.003 | 0.014 |
| Q8.8 | 0.048 | 0.375 | 0.024 | 0.000 | -0.006 | 0.024 |
| Q8.14 | 0.032 | 0.251 | 0.016 | 0.000 | -0.004 | 0.016 |
| Q8.5 | 0.003 | 0.005 | 0.075 | 0.012 | 0.003 | 0.041 |
| Q8.15 | 0.004 | 0.006 | 0.082 | 0.013 | 0.003 | 0.045 |
| Q8.17 | 0.005 | 0.007 | 0.104 | 0.016 | 0.004 | 0.057 |
| Q8.20 | 0.004 | 0.006 | 0.084 | 0.013 | 0.004 | 0.046 |
| Q8.23 | 0.003 | 0.005 | 0.066 | 0.010 | 0.003 | 0.036 |
| Q8.26 | 0.004 | 0.006 | 0.091 | 0.014 | 0.004 | 0.050 |
| Q8.2 | 0.005 | 0.000 | 0.013 | 0.035 | 0.003 | 0.003 |
| Q8.11 | 0.008 | 0.000 | 0.019 | 0.052 | 0.004 | 0.004 |
| Q8.13 | 0.008 | 0.000 | 0.020 | 0.053 | 0.004 | 0.004 |
| Q8.19 | 0.014 | 0.000 | 0.034 | 0.091 | 0.007 | 0.007 |
| Q8.24 | 0.017 | 0.000 | 0.042 | 0.114 | 0.009 | 0.009 |
| Q8.25 | 0.010 | 0.000 | 0.025 | 0.069 | 0.006 | 0.005 |
| Q8.1 | 0.006 | -0.002 | 0.004 | 0.003 | 0.044 | 0.002 |
| Q8.7 | 0.007 | -0.002 | 0.006 | 0.005 | 0.057 | 0.002 |
| Q8.21 | 0.009 | -0.003 | 0.007 | 0.006 | 0.073 | 0.003 |
| Q8.22 | 0.011 | -0.004 | 0.009 | 0.007 | 0.090 | 0.003 |
| Q8.27 | 0.011 | -0.004 | 0.009 | 0.007 | 0.090 | 0.003 |
| Q8.28 | 0.016 | -0.005 | 0.012 | 0.010 | 0.124 | 0.005 |
| Q8.30 | 0.001 | 0.003 | 0.026 | 0.002 | 0.001 | 0.065 |
| Q8.29 | 0.001 | 0.004 | 0.034 | 0.002 | 0.001 | 0.086 |
| Q8.16 | 0.002 | 0.006 | 0.045 | 0.003 | 0.001 | 0.114 |
| Q8.10 | 0.002 | 0.007 | 0.057 | 0.003 | 0.002 | 0.144 |
| Q8.9 | 0.002 | 0.006 | 0.044 | 0.003 | 0.001 | 0.110 |
| Q8.6 | 0.002 | 0.006 | 0.045 | 0.003 | 0.001 | 0.112 |

**S4 Table:** CFA Factor loadings for the DOSPERT survey. DOSPERT question numbers are given in the first column (Q8 is risk perception) and the factor loadings given under the six DOSPERT dimension headings.
