# Supplementary material for: Characterizing altruistic motivation in potential volunteers for SARS-CoV-2 challenge trials
Source: PLoS One. 2022 Nov 2;17(11):e0275823. doi: 10.1371/journal.pone.0275823 (PMC9629635; doi:10.1371/journal.pone.0275823)
Supplement: S5 Table — (DOCX) [file pone.0275823.s009.docx]

**S5 Table. DOSPERT Factor loadings: Perceived Benefits**

| Factor Score Weights - DOSPERT Perceived Benefits | | |  |  |  |  |
| --- | --- | --- | --- | --- | --- | --- |
|  | FinanceInvest | FinanceGamble | HealthSafety | Recreation | Social | Ethical |
| Q9.4 | 0.090 | 0.009 | -0.003 | 0.005 | 0.009 | 0.005 |
| Q9.12 | 0.238 | 0.023 | -0.008 | 0.012 | 0.023 | 0.012 |
| Q9.18 | 0.220 | 0.022 | -0.008 | 0.011 | 0.022 | 0.011 |
| Q9.3 | 0.024 | 0.195 | 0.009 | -0.001 | -0.004 | 0.014 |
| Q9.8 | 0.038 | 0.313 | 0.014 | -0.001 | -0.007 | 0.023 |
| Q9.14 | 0.037 | 0.304 | 0.013 | -0.001 | -0.007 | 0.022 |
| Q9.5 | -0.004 | 0.004 | 0.075 | 0.007 | 0.001 | 0.030 |
| Q9.15 | -0.003 | 0.003 | 0.046 | 0.004 | 0.001 | 0.019 |
| Q9.17 | -0.011 | 0.011 | 0.188 | 0.017 | 0.004 | 0.077 |
| Q9.20 | -0.011 | 0.011 | 0.183 | 0.016 | 0.004 | 0.075 |
| Q9.23 | -0.006 | 0.006 | 0.096 | 0.009 | 0.002 | 0.039 |
| Q9.26 | -0.004 | 0.004 | 0.072 | 0.006 | 0.001 | 0.029 |
| Q9.2 | 0.003 | 0.000 | 0.003 | 0.044 | 0.004 | 0.001 |
| Q9.11 | 0.006 | 0.000 | 0.006 | 0.086 | 0.008 | 0.001 |
| Q9.13 | 0.007 | 0.000 | 0.007 | 0.108 | 0.010 | 0.001 |
| Q9.19 | 0.010 | 0.000 | 0.010 | 0.150 | 0.014 | 0.002 |
| Q9.24 | 0.010 | 0.000 | 0.011 | 0.160 | 0.015 | 0.002 |
| Q9.25 | 0.006 | 0.000 | 0.007 | 0.098 | 0.009 | 0.001 |
| Q9.1 | 0.006 | -0.001 | 0.001 | 0.005 | 0.050 | 0.001 |
| Q9.7 | 0.013 | -0.002 | 0.002 | 0.010 | 0.099 | 0.001 |
| Q9.21 | 0.012 | -0.002 | 0.001 | 0.009 | 0.092 | 0.001 |
| Q9.22 | 0.016 | -0.003 | 0.002 | 0.012 | 0.124 | 0.001 |
| Q9.27 | 0.011 | -0.002 | 0.001 | 0.009 | 0.085 | 0.001 |
| Q9.28 | 0.016 | -0.003 | 0.002 | 0.012 | 0.119 | 0.001 |
| Q9.30 | 0.003 | 0.004 | 0.016 | 0.001 | 0.001 | 0.045 |
| Q9.29 | 0.008 | 0.010 | 0.039 | 0.002 | 0.001 | 0.113 |
| Q9.16 | 0.012 | 0.014 | 0.056 | 0.002 | 0.002 | 0.163 |
| Q9.10 | 0.008 | 0.010 | 0.039 | 0.002 | 0.001 | 0.114 |
| Q9.9 | 0.006 | 0.007 | 0.028 | 0.001 | 0.001 | 0.081 |
| Q9.6 | 0.007 | 0.008 | 0.031 | 0.001 | 0.001 | 0.090 |

**S5 Table:** CFA Factor loadings for the DOSPERT survey. DOSPERT question numbers are given in the first column (Q9 is perceived benefits) and the factor loadings given under the six DOSPERT dimension headings.
