# Supplementary material for: Characterizing altruistic motivation in potential volunteers for SARS-CoV-2 challenge trials
Source: PLoS One. 2022 Nov 2;17(11):e0275823. doi: 10.1371/journal.pone.0275823 (PMC9629635; doi:10.1371/journal.pone.0275823)
Supplement: S6 Table — (DOCX) [file pone.0275823.s010.docx]

## **S6 Table. DOSPERT Scale Factors and corresponding questions in the survey**

| **DOSPERT Scale Factor** |
| --- |
|  |
| Ethical (6,9,10,16,29,30) |
| Financial-Investing (4,12,18) |
| Financial-Gambling (3,8,14) |
| Health/Safety (5,15,17,20,23,26) |
| Recreational (2,11,13,19,24,25) |
| Social (1,7,21,22,27,28) |

**S6 Table:** Details of the questions on the 30-question DOSPERT survey corresponding to the original six DOSPERT factors. Risk taking likelihood, risk perception, and perceived benefits all use the same factor design.
