# Supplementary material for: Characterizing altruistic motivation in potential volunteers for SARS-CoV-2 challenge trials
Source: PLoS One. 2022 Nov 2;17(11):e0275823. doi: 10.1371/journal.pone.0275823 (PMC9629635; doi:10.1371/journal.pone.0275823)
Supplement: S7 Table — (DOCX) [file pone.0275823.s011.docx]

**S7 Table. Comparisons of DOSPERT risk attitude component scores.**

|  | **Volunteer Group**  **(n=1669)** | | **Control**  **(n=770)** | | **p-value** | **η2** |
| --- | --- | --- | --- | --- | --- | --- |
| ***Risk taking likelihood*** | ***Mean (SE)*** | ***95% CI*** | ***Mean (SE)*** | ***95% CI*** |  |  |
| Ethical | 1.457 (0.023) | 1.412, 1.501 | 2.335 (0.036) | 2.265, 2.405 | < 0.001 | 0.136 |
| Financial - Investment | 2.258 (0.020) | 2.220, 2.297 | 2.255 (0.031) | 2.195, 2.315 | 0.928 | - |
| Financial - Gambling | 1.402 (0.028) | 1.347, 1.456 | 2.338 (0.043) | 2.253, 2.423 | < 0.001 | 0.107 |
| Health/Safety | 2.427 (0.024) | 2.380, 2.474 | 2.944 (0.037) | 2.871, 3.017 | < 0.001 | 0.047 |
| Recreational | 2.204 (0.022) | 2.161, 2.246 | 1.870 (0.034) | 1.804, 1.937 | < 0.001 | 0.024 |
| Social | 2.587 (0.012) | 2.563, 2.610 | 2.139 (0.019) | 2.103, 2.176 | < 0.001 | 0.129 |
| ***Risk perception*** | ***Mean (SE)*** | ***95% CI*** | ***Mean (SE)*** | ***95% CI*** | **p-value** | **η2** |
| Ethical | 4.931 (0.028) | 4.876, 4.985 | 4.910 (0.043) | 4.825, 4.995 | 0.700 | - |
| Financial - Investment | 3.543 (0.021) | 3.502, 3.583 | 3.632 (0.032) | 3.569, 3.696 | 0.028 | 0.002 |
| Financial - Gambling | 5.232 (0.036) | 5.162, 5.301 | 4.927 (0.056) | 4.818, 5.037 | < 0.001 | 0.008 |
| Health/Safety | 4.816 (0.026) | 4.765, 4.866 | 4.954 (0.040) | 4.875, 5.033 | 0.007 | 0.003 |
| Recreational | 2.403 (0.016) | 2.372, 2.434 | 2.694 (0.025) | 2.646, 2.742 | < 0.001 | 0.035 |
| Social | 1.620 (0.014) | 1.593, 1.648 | 1.987 (0.022) | 1.944, 2.031 | < 0.001 | 0.066 |
| ***Perceived benefits*** | ***Mean (SE)*** | ***95% CI*** | ***Mean (SE)*** | ***95% CI*** | **p-value** | **η2** |
| Ethical | 1.874 (0.023) | 1.828, 1.920 | 2.620 (0.036) | 2.548, 2.691 | < 0.001 | 0.097 |
| Financial - Investment | 2.651 (0.023) | 2.606, 2.696 | 2.711 (0.036) | 2.641, 2.782 | 0.185 | - |
| Financial - Gambling | 1.962 (0.031) | 1.901, 2.023 | 2.824 (0.049) | 2.729, 2.919 | < 0.001 | 0.075 |
| Health/Safety | 1.619 (0.023) | 1.575, 1.663 | 2.349 (0.035) | 2.280, 2.419 | < 0.001 | 0.100 |
| Recreational | 2.504 (0.026) | 2.454, 2.554 | 2.361 (0.040) | 2.283, 2.440 | 0.005 | 0.003 |
| Social | 2.908 (0.018) | 2.873, 2.943 | 2.629 (0.028) | 2.574, 2.685 | < 0.001 | 0.025 |
| *Comparison F-tests were performed for the volunteer and control groups on all three separate scale scores for the DOSPERT risk assessment test, controlled for age, income, country of residence, education level and gender (using an ANCOVA model).* | | | | | | |

**S7 Table:** Covariates for age, income, education level, gender, and US residency were included to control for the potential role of demographic differences between volunteer and control respondents. Relative to controls, volunteers demonstrated greater risk aversion in the domains of ethics, gambling, and health and safety, and were more risk seeking than controls with respect to recreational and social behaviors. Volunteers showed higher risk perception than controls in financial-gambling, but lower risk perception than controls in financial-investment, health and safety, recreational, and social activities. However, most of the effects were trivial or small. Volunteers also showed higher perceived benefits than controls in recreational and social activities (albeit with very small effect sizes), but lower perceived benefits than controls in ethical, financial-gambling, and health and safety activities (with much larger effect sizes).
